# Human Recombinant Alkaline Phosphatase (Ilofotase Alfa) Protects Against Kidney Ischemia-Reperfusion Injury in Mice and Rats Through Adenosine Receptors

**DOI:** 10.3389/fmed.2022.931293

**Published:** 2022-07-28

**Authors:** Diane L. Rosin, J. Perry Hall, Shuqiu Zheng, Liping Huang, Silvia Campos-Bilderback, Ruben Sandoval, Andrea Bree, Kevin Beaumont, Emily Miller, Jennifer Larsen, Ghazal Hariri, Neelu Kaila, Iain M. Encarnacion, Jeremy D. Gale, Andrea van Elsas, Bruce A. Molitoris, Mark D. Okusa

**Affiliations:** ^1^Department of Pharmacology, University of Virginia, Charlottesville, VA, United States; ^2^Inflammation and Immunology Research Unit, Pfizer Inc., Cambridge, MA, United States; ^3^Division of Nephrology, Center for Immunity, Inflammation and Regeneration, University of Virginia, Charlottesville, VA, United States; ^4^Division of Nephrology, Department of Medicine, Indiana University School of Medicine, Indiana Center for Biological Microscopy, Roudebush VA Medical Center, Indianapolis, IN, United States; ^5^BioMedicine Design, Pfizer Inc., Cambridge, MA, United States; ^6^BioMedicine Design, Pfizer Inc., Groton, CT, United States; ^7^Early Clinical Development, Pfizer Inc., Groton, CT, United States; ^8^Drug Product Development, Pfizer Inc., Cambridge, MA, United States; ^9^Medicinal Chemistry, Pfizer Inc., Cambridge, MA, United States; ^10^AM-Pharma B.V., Utrecht, Netherlands

**Keywords:** acute kidney injury, adenosine, ATP, IRI, CD73, alkaline phosphatase, recAP

## Abstract

Adenosine triphosphate (ATP) released from injured or dying cells is a potent pro-inflammatory “danger” signal. Alkaline phosphatase (AP), an endogenous enzyme that de-phosphorylates extracellular ATP, likely plays an anti-inflammatory role in immune responses. We hypothesized that ilofotase alfa, a human recombinant AP, protects kidneys from ischemia-reperfusion injury (IRI), a model of acute kidney injury (AKI), by metabolizing extracellular ATP to adenosine, which is known to activate adenosine receptors. Ilofotase alfa (iv) with or without ZM241,385 (sc), a selective adenosine A_2A_ receptor (A_2A_R) antagonist, was administered 1 h before bilateral IRI in WT, A_2A_R KO (*Adora2a^–/–^*) or *CD73^–/–^* mice. In additional studies recombinant alkaline phosphatase was given after IRI. In an AKI-on-chronic kidney disease (CKD) ischemic rat model, ilofotase alfa was given after the three instances of IRI and rats were followed for 56 days. Ilofotase alfa in a dose dependent manner decreased IRI in WT mice, an effect prevented by ZM241,385 and partially prevented in *Adora2a^–/–^* mice. Enzymatically inactive ilofotase alfa was not protective. Ilofotase alfa rescued *CD73^–/–^* mice, which lack a 5′-ectonucleotidase that dephosphorylates AMP to adenosine; ZM241,385 inhibited that protection. In both rats and mice ilofotase alfa ameliorated IRI when administered after injury, thus providing relevance for therapeutic dosing of ilofotase alfa following established AKI. In an AKI-on-CKD ischemic rat model, ilofotase alfa given after the third instance of IRI reduced injury. These results suggest that ilofotase alfa promotes production of adenosine from liberated ATP in injured kidney tissue, thereby amplifying endogenous mechanisms that can reverse tissue injury, in part through A_2A_R-and non-A_2A_R-dependent signaling pathways.

## Introduction

Acute kidney injury (AKI) can result from a variety of causes and is associated with high morbidity and mortality (1). The incidence of AKI is 10–15% in hospital patients and as high as 60% in ICU patients (2), and sepsis is the most common cause of AKI in these ICU patients (1, 2). AKI is often associated with multi-organ dysfunction or sepsis, and in the ICU setting AKI-associated mortality rates can be 30–70% (3). Recovery from AKI leaves patients at increased risk for developing or exacerbating chronic kidney disease (CKD) or progressing to end stage kidney disease (4, 5). AKI is a global health concern (6), and searching for therapeutic targets is critical to uncovering greatly needed approaches for treatment or prevention of AKI and its progression to CKD.

Alkaline phosphatase (AP) is one candidate molecule with the potential to inhibit multiple inflammatory signals relevant to sepsis, sepsis-associated AKI, and other forms of AKI. AP isoforms exert detoxifying effects through dephosphorylation of a variety of soluble inflammatory mediators and other deleterious compounds. In animal models of sepsis (e.g., LPS- or *Escherichia coli*-induced sepsis and cecal ligation puncture-induced sepsis), administration of AP from human placenta (7, 8), calf intestine (9), and bovine intestine (10, 11) attenuated inflammatory responses, reduced toxicity, and increased survival rates. The protective effect of exogenous AP in sepsis models may derive from its ability to dephosphorylate and thereby detoxify LPS (7, 12, 13).

Therapeutic efficacy of bovine intestinal AP (BIAP) for treatment of sepsis and sepsis-associated AKI was investigated in two small Phase 2 clinical trials and revealed improved kidney function and better survival (14, 15). To expand clinical trials but avoid possible adverse effects of animal-derived AP, a human recombinant AP, ilofotase alfa (previously referred to as recAP), with high stability and enzymatic selectivity was developed (16). In a randomized Phase 2a/2b clinical trial of 301 critically ill patients with sepsis-associated AKI (the STOP-AKI trial) recAP did not significantly improve kidney function in the 7-day period immediately following treatment, however at 21–28 days creatinine clearance was higher in the treatment group than in placebo-treated patients and mortality was lower in the recAP-treated group (17). Expanded investigation of the therapeutic potential of ilofotase alfa is ongoing in a global Phase 3 pivotal trial (NCT04411472), which will be the largest clinical trial in sepsis-associated AKI conducted to date.

In addition to preclinical studies evaluating AP for efficacy in treating sepsis and sepsis-associated AKI [previously reviewed (18, 19)], protective effects of AP, and recAP in particular, have been examined in other animal models of kidney injury. In an ischemia-reperfusion injury (IRI) model of AKI in rats, recAP improved kidney hemodynamics and microvascular oxygenation and reduced kidney injury and inflammation (20). RecAP reduced inflammation elicited by antimycin A-induced mitochondrial dysfunction in conditionally immortalized human proximal tubule epithelial cells (21). Both BIAP and recAP protected rat kidneys from cisplatin- and gentamicin-induced AKI (22).

Studies on the mechanism(s) underlying the therapeutic efficacy of AP have mostly focused on its ability to inactivate damage-associated molecular patterns (DAMPs) and pathogen-associated molecular patterns (PAMPs), thereby reducing inflammation. However, direct kidney protective effects during injury and disease may involve other mechanisms and other endogenous AP substrates. Adenosine triphosphate (ATP), released from damaged and dying cells into the extracellular space during tissue injury, can bind to P2 receptors and produce detrimental effects or can be a substrate for extracellular conversion to adenosine (23). APs, and recAP in particular, are capable of the sequential enzymatic breakdown and dephosphorylation of extracellular ATP to yield adenosine (16, 19, 24), but ATP can also be metabolized by other ectonucleotidases. CD73, an ecto-5′-nucleotidase that converts AMP to adenosine, is critical for protection against mouse kidney IRI, and IRI is exacerbated in *CD73^–/–^* mice subjected to sub-threshold ischemic conditions (25). This phenotype is likely a result of deficiencies in adenosine production and anti-inflammatory effects of adenosine receptor (AR) signaling in *CD73^–/–^* animals. Thus, *CD73^–/–^* mice could be a useful model system for implicating ATP, ADP, and AMP dephosphorylation in recAP’s mechanism of action during kidney IRI.

We (26–32) and others (33, 34) have shown previously that adenosine A_2A_ receptor (A_2A_R) activation inhibits kidney injury and reduces inflammation in kidney IRI. For the present manuscript, we hypothesized that recAP’s ability to metabolize released extracellular ATP, ADP, or AMP to adenosine (16, 19, 24) leads to kidney protective effects through activation of ARs. We demonstrate prophylactic and therapeutic efficacy of human ilofotase alfa against AKI in mice and rats and in protecting rats from AKI on a background of CKD. Using the adenosine A_2A_R antagonist ZM241,385, A_2A_R KO (*Adora2a^–/–^*) mice and *CD73^–/–^* mice, we show that AR signaling is a component of recAP’s mechanism of action and that recAP restores the deficit in adenosine production and anti-inflammatory AR signaling in *CD73^–/–^* mice. Taken together, these results implicate the augmentation of adenosine signaling as a key component of the mechanism of action of ilofotase alfa in rodent kidney IRI and provide additional support for the potential of ilofotase alfa as a treatment option for AKI.

## Materials and Methods

### Mice and Ischemia-Reperfusion Injury Surgery

We used male C57BL/6 (WT) mice (∼20–24 g, 8–12 weeks of age, National Cancer Institute, Frederick, MD, United States) and *Adora2a^–/–^* mice (A_2A_R KO mice; congenic on C57BL/6; originally provided by Chen et al. (35) and now available from The Jackson Laboratory, stock no. 010685) and their littermate controls. Mice were anesthetized with a mixture (ip) of ketamine (120 mg/kg) and xylazine (12 mg/kg) and were subjected to bilateral flank incisions. Bilateral ischemia was induced by exposing the kidney artery and vein and cross-clamping for 26 min, then clamps were released as described before (36) and kidneys were allowed to reperfuse. The kidney artery/vein was exposed but not clamped in sham-operated mice. During the surgery, mouse core temperature was maintained at 34–36°C with a TR-200 Heating Pad System (Fine Science Tools); during the recovery and reperfusion period (18–24 h), mice were housed in a warming incubator with ambient temperature at 30–32°C. At the termination of experiments, mice were anesthetized and blood was collected from the retroorbital plexus for plasma creatinine measurements. Kidneys were collected and portions were fixed in 10% buffered formalin (Fisher Scientific, Suwanee, GA, United States) overnight for histological staining or snap frozen in liquid nitrogen and saved for protein, mRNA, or adenosine analyses.

For other experiments male B6.129S1-*Nt5e^TM1Lft^*/J (*^CD73–/–^*) mice (∼20–24 g, 8–12 weeks of age; on C57BL/6J background) were originally provided by Linda Thompson (Oklahoma Medical Research Foundation, Oklahoma City, OK, United States) and were also purchased from The Jackson Laboratory, stock no. 018986; Jackson C57BL/6J WT mice and CD73 wild-type littermates (*CD73*^+/+^) were used as controls. An ischemic time of 20 min was used for these experiments based on prior studies (25).

Surgeries or drug treatments were always performed in a dedicated surgical/animal procedure room between the hours of 10:00 a.m. and 2:00 p.m. so that blood collection and euthanasia could also occur during this same time of day on the desired number of days after treatment. Animals were housed in the vivarium and allowed to acclimate before procedures and between procedures in intervening periods of time greater than 24 h with free access to food and water and with a standard light/dark cycle. In all experiments, animals were randomized to treatment (vehicle and drug dosing) and surgery (sham vs. IRI), and the experimenter was blinded for treatment (solutions had coded labels), creatinine assay and histological scoring. Sets of control and treatment groups were always performed on the same day.

### Preparation and Administration of Drugs

Stock solutions of human recAP (batch #119A15-10; aka ilofotase alfa), prepared in a stock vehicle of 20 mM sodium citrate/250 mM D-sorbitol/2 mM MgCl_2_/50 μm ZnCl_2_, pH 7.0, were stored at −80°C and diluted (1:35) immediately before use with sterile saline to yield a working solution for the highest dose of recAP (2,000 U/kg); serial dilutions were made with a comparable dilution of the stock vehicle (1:35 in saline) to yield working solutions for injections (i.v.) with the identical composition of vehicle components. Final vehicle composition of all working recAP solutions and the solution that was used as the administered vehicle control was 0.57 mM sodium citrate/7.14 mM D-sorbitol/57 μM MgCl_2_/1.43 μm ZnCl_2_, pH 7.0.

A recombinant catalytically inactive mutant form of human recAP (mut-recAP, aka S92A recAP), which has been used previously (37), was generated by subcloning the sequence (containing the S92A mutated sequence) into an expression vector and transiently expressing the protein in CHO cells. The protein was isolated and purified (by anion exchange and gel filtration). Enzyme activity [the amount of enzyme causing the hydrolysis of 1 μM p-nitrophenyl phosphate (pNPP) per minute at 25°C and pH 9.6] was confirmed to be undetectable (∼0 U/mg protein). Catalytically inactive mut-recAP (prepared in the same vehicle as enzymatically active recAP) was stored at −80°C and diluted immediately before use with sterile saline to achieve the same molar concentration as the solution used for the highest dose of enzymatically active recAP (2,000 U/ml).

For experiments in WT mice, the AR antagonist, ZM241,385 was prepared as a stock solution in DMSO (stored at −20°C). For experiments in *CD73^–/–^* mice and for pharmacokinetic studies, ZM241,385 (Synnovator, Inc., Durham, NC, United States; cat. #139180-30-6) was prepared immediately before use by dissolving in PEG-400 followed by slow sequential addition of Cremophor EL then methylcellulose to yield final proportions of 15% (v/v) Cremophor EL (Calbiochem, San Diego, CA, United States)/30% (v/v) PEG-400 (Sigma-Aldrich)/55% (v/v) 0.5% methylcellulose in DI water. Working solutions of ZM241,385 and vehicle were prepared immediately before use by dilution of stock solutions with sterile saline.

ZM241,385 was administered subcutaneously in mice immediately before recAP; recAP and mut-recAP were administered intravenously 1 h before IRI surgery, except as noted (0.5, 1, 4 h after IRI) in some experiments.

Blood was collected under isoflurane anesthesia by retro-orbital bleeding after 24 h of kidney reperfusion. Mice were euthanized (by cervical dislocation under anesthesia immediately after collecting blood), and kidneys were harvested.

### Pharmacokinetic Studies of ZM241,385

C57BL/6 female mice, approximately 6–8 weeks of age (Charles River Labs, Wilmington, MA, United States; *n* = 3 per treatment group), were used. ZM241,385 was administered (22.3, 92.3, or 180.8 mg/kg; s.c.) in a volume of 10 ml/kg. Mice were euthanized 1, 4, 8, and 24 h later, and blood was collected by cardiac puncture. Plasma was separated by centrifugation and stored at −80°C until analysis.

Plasma samples were analyzed by liquid chromatography–tandem mass spectrometry (LC-MS/MS). Analytical standards for each analyte were prepared by solubilizing powder stocks into 1:1 DMSO:ACN and standard curves were prepared in naïve animal plasma. Samples were extracted using protein precipitation with ∼5 volumes of an internal standard cocktail (tolbutamide at 5 ng/ml) in acetonitrile. Sample blocks were vortexed for 30 s and centrifuged at 3,000 rpm for 5′ and ∼50 μl of the supernatant was diluted into three volumes of water containing 0.1% formic acid for injection onto the LC-MS.

Chromatography was performed on a Waters Acquity iClass UPLC System (Milford, MA, United States). The autosampler and column were kept at 10 and 40°C, respectively. Separation was achieved with an Acquity UPLC HSS T3 column (2.1 × 50 mm, 1.8 μm) and a gradient of 0.1% formic acid in water (Mobile Phase A) and 0.1% formic acid in acetonitrile (Mobile Phase B) at a flow rate of 0.600 ml/min. An initial mobile phase composition of 5% B was ramped to 95% over 2 min, held at 95% for 0.3 min, and then returned to initial 5% B for re-equilibration.

Data were collected on an AB Sciex API5500 mass spectrometer (Foster City, CA, United States) using negative Turbo IonSpray™ electrospray ionization (ESI) and multiple reaction monitoring (MRM) mode. MRM transitions for the analyte, including DP and CE, were as follows: PF-01192544 336→174 DP: −62 CE: −33. Tolbutamide was used as the internal standard for PF-01192544 (MRM transitions = 269→170 DP: −60 CE: −30). Data acquisition and processing was carried out with Analyst software version 1.6.2 (Applied Biosystems/MDS Sciex, Canada).

### Assessment of Kidney Function and Histology

Plasma creatinine was measured by enzymatic assay according to the manufacturer’s instructions (Diazyme Laboratories, Poway, CA, United States) but using twice the recommended sample volume; the accuracy of this method has been verified by LC-MS (38, 39). Portions of kidney were fixed in 4% paraformaldehyde in 0.1 M sodium phosphate buffer, pH 7.0; paraffin sections were cut and stained with hematoxylin and eosin (H&E). Photomicrographs were acquired using an MBF Bioscience and Zeiss AxioImager Z1/Apotome microscope system using StereoInvestigator software (MBF Bioscience, Williston, VT, United States).

### Outer Medulla Tubular Damage Scoring

Tubular necrosis in the kidney outer medulla was scored in H&E-stained kidney sections by an MBF Bioscience and Zeiss AxioImager Z1/Apotome microscope system for stereology and tissue morphology in a blinded fashion (slides were identified by a code that was not revealed to the person scoring the samples) by a method described previously (36) and using StereoInvestigator software (MBF Bioscience). The results represent the percentage of total surface area of the cortex and outer medullary region (region defined by an outline and area of the region calculated by the software) occupied by injured tubules (0–100% of the region). Acute tubular injury included tubule dilation and denudation, tubular epithelial denucleation, and cast formation. The following parameters were defined: counting frame, 400 × 400 μm; sample grid, 700 × 700 μm; grid spacing, 75 μm. These values were determined empirically such that adequate numbers of sample sites were visited and adequate numbers of markers (indicating acute tubular injury) were acquired, in keeping with accepted counting rules for stereology. A total of 280 ± 10 (mean ± SE) grid sites were evaluated per section; the sampling fraction was 32% of a total average area of 10.70 ± 0.43 μm × 10^6^ μm for each kidney section.

### Models of Acute Kidney Injury and Acute Kidney Injury on Chronic Kidney Disease in Rats

Isoflurane was used as anesthetic for all studies in rats. Male Sprague-Dawley rats, 220–250 g (Harlan), were used in a recAP dose escalation study (50–2,000 U/Kg, i.v.) in a rat unilateral kidney clamp model of ischemic AKI (30 min unilateral ischemia with simultaneous right nephrectomy followed by clamp release and a 24-h period of reperfusion). Sterile saline or the dose of recAP was administered i.v. over a period of 1 min just after the 30 min kidney pedicle clamp was removed, and plasma creatinine was measured at the end of the 24 h period of reperfusion, as previously reported (40). Munich Wistar Fromter (MWF) male and female rats, 180–260 g (from breeding colony; a generous gift of Roland Blantz), were used for the repetitive AKI-associated uninephrectomized proteinuric CKD model study (AKI on CKD model). For the repetitive AKI model, rats 8–10 weeks of age underwent a simultaneous right nephrectomy and 45 min left kidney pedicle clamp procedure to induce AKI, as described previously (40). Four weeks later they underwent a second pedicle clamp for 40 min. To test the effect of recAP in this aged and proteinuric CKD model, rats were randomized to vehicle or drug groups and underwent a third pedicle clamp for 30 min on day 56 of the study (16–18 weeks of age) followed by treatment with vehicle (sterile saline) or recAP (1,000 U/Kg, i.v.) at 0 (immediately after releasing the clamp), 24 and 48 h after ischemia. A control group of rats had right nephrectomy and sham IRI surgery with no clamping. Animals were fed a standard diet throughout. Twenty four-hour urine samples were collected over sodium azide in metabolic cages and the volume of collected urine was recorded. Creatinine concentration was measured in blood plasma and in urine as previously described (40). Glomerular filtration rate (GFR) was calculated using 24 h urinary creatinine values and serum creatinine concentration values. Urine protein concentration was determined according to Bradford’s method (Thermo Scientific, Rockford, IL, United States), with bovine serum albumin as standard. Rats were euthanized by pentobarbital overdose followed by cervical dislocation.

### Statistics

Mice: data were analyzed using one-way or two-way analysis of variance (ANOVA) with a significant difference defined as *P* < 0.05. Means were compared by *post hoc* multiple-comparison test (Tukey’s), and all values are presented as mean ± SEM and as individual values in dot plots. Analyses were performed with GraphPad Prism version 6 (GraphPad Software Inc., La Jolla, CA, United States). Rats: a one-sided Student’s *t*-test was used for 2 group comparisons.

## Results

### Ilofotase Alfa Produced a Dose-Dependent Decrease in Kidney Injury in WT Mice

**FIGURE 1:**
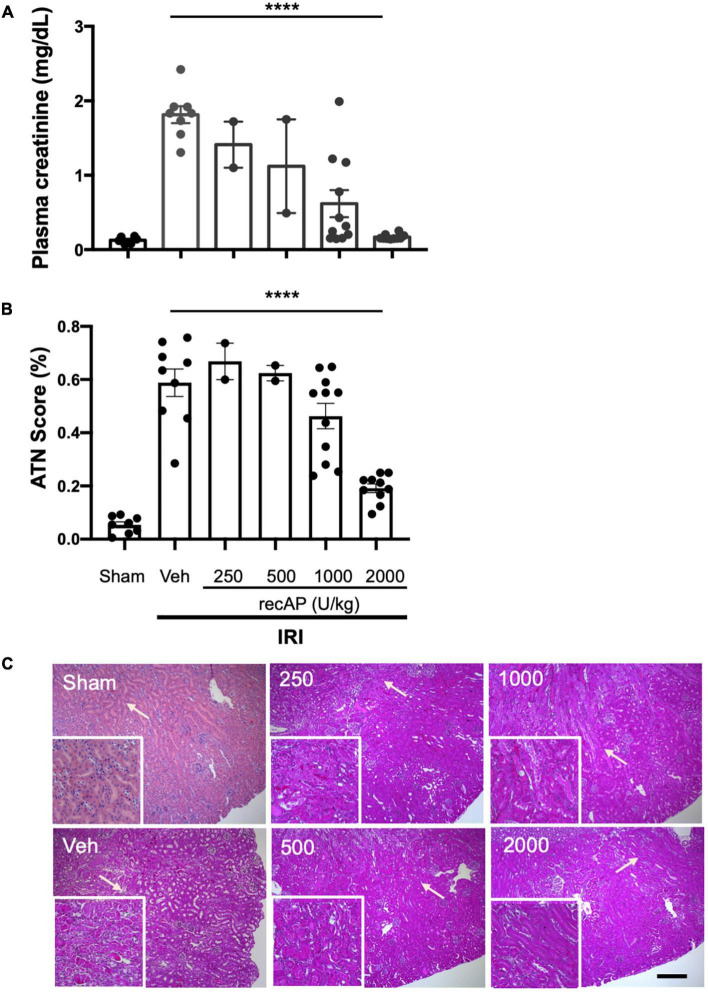
Human recombinant alkaline phosphatase (recAP, aka ilofotase alfa) protects male WT mice from ischemia-reperfusion injury (IRI) in a dose-dependent fashion. Vehicle (veh) or ilofotase alfa (recAP) was administered intravenously 1 h before 26 min bilateral kidney ischemia, and mice were euthanized after 24 h of reperfusion. Control mice were subjected to sham surgery (no ischemic clamp). **(A)** Plasma creatinine measured at 24 h. **(B)** Quantification of acute tubular necrosis (ATN) in the cortex and outer medulla (injury expressed as % of total kidney section surface area; 0–100%) was scored stereologically from hematoxylin and eosin-(H&E) stained kidney sections. **(C)** Representative histology of H&E-stained sections (same mice as for plasma creatinine). Area depicted in main panels includes cortex (from edge of tissue section) through outer medulla and in some cases outer edge of inner medulla. Scale bar: 200 μm in main panels and 100 μm in insets (outer medulla). Areas indicated by arrows are magnified in the insets. Veh, vehicle. Values for individual mice and the mean and SE are shown in **(A,B)**. ^*⁣*⁣**^P < 0.0001 by one-way ANOVA. *n* = 2–10.

Choosing doses based on prior studies of recAP in models of sepsis (20, 37), we evaluated the ability of ilofotase alfa to protect kidneys of WT mice from IRI, a common model of AKI. Plasma creatinine was elevated after 26 min of ischemia and 24 h of reperfusion in vehicle-treated mice compared to mice subjected to sham surgery. In a pilot study, doses of ilofotase alfa of 250 and 500 U/kg (i.v.) given 1 h before IRI produced small or variable degrees of protection and were not included in subsequent experiments. An ilofotase alfa dose of 1,000 U/kg afforded partial protection, and 2,000 U/kg maximally protected kidneys from injury (Figure 1A). Stereological scoring of injury (acute tubular necrosis, ATN; using same mice as for plasma creatinine; Figure 1B) in H&E sections (Figure 1C) yielded results comparable to plasma creatinine.

### Enzymatic Activity of Ilofotase Alfa Is Required for Kidney Protection

**FIGURE 2:**
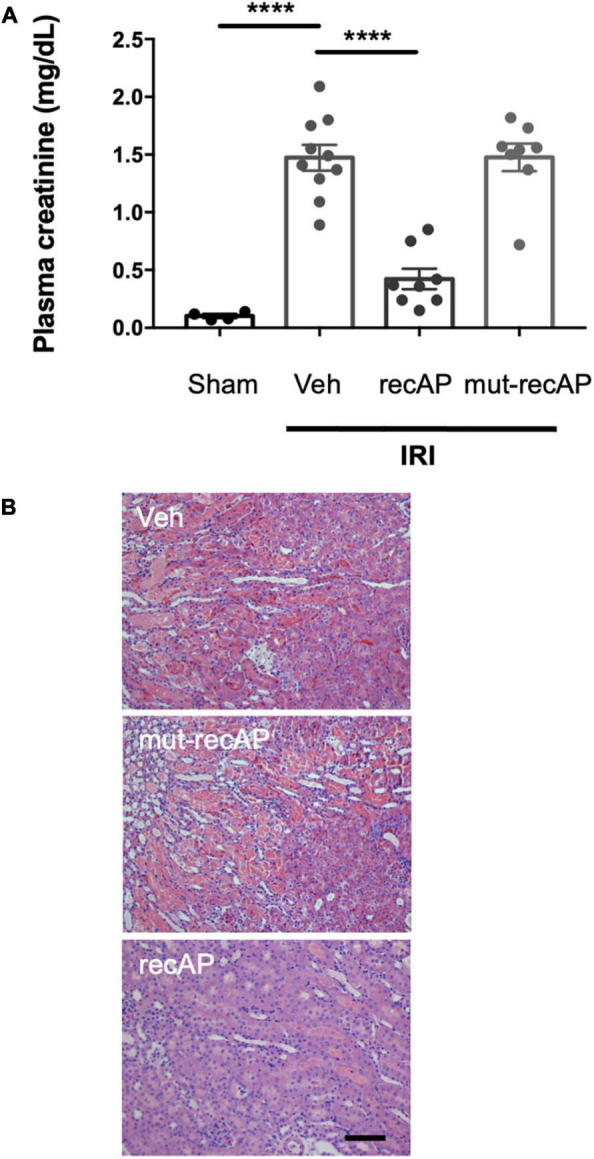
Protection from IRI requires enzymatic activity of ilofotase alfa. Male WT mice received ilofotase alfa (recAP, 2,000 U/kg, i.v.) or an equivalent dose (based on calculated mg/kg) of a catalytically inactive mutant recAP (mut-recAP) 1 h before 26 min bilateral kidney ischemia and 24 h of reperfusion. **(A)** Plasma creatinine. **(B)** Representative histology of outer medulla from H&E-stained sections. Veh, vehicle. Scale bar: 100 μm. Error bars represent mean ± SEM. ****P < 0.0001 by one-way ANOVA. *n* = 4–10.

To evaluate whether the enzymatic activity of ilofotase alfa is needed for the protection from IRI, we administered a catalytically inactive mutant form of the enzyme (mut-recAP) carrying a single S92A mutation. With equimolar concentrations of the enzymatically active and mutant proteins, only the active form of the enzyme can dephosphorylate ATP (37). In the current study, the most effective dose of ilofotase alfa (2,000 U/kg) and equivalent amount of mut-recAP (based on calculated mg recombinant protein/kg) were administered (i.v.) to mice 1 h prior to IRI (26 min of ischemia followed by 24 h of reperfusion). Only the enzymatically active form of ilofotase alfa protected kidneys from IRI. Plasma creatinine (Figure 2A) and ATN (Figure 2B; see also histology, Supplementary Figure 1) in mice receiving mut-recAP were not different from vehicle. These data are consistent with, though not proof of, a critical role for ATP dephosphorylation in the protective effect of ilofotase alfa in kidney IRI.

### Adenosine Receptors Are Important for Ilofotase Alfa Protection

**FIGURE 3:**
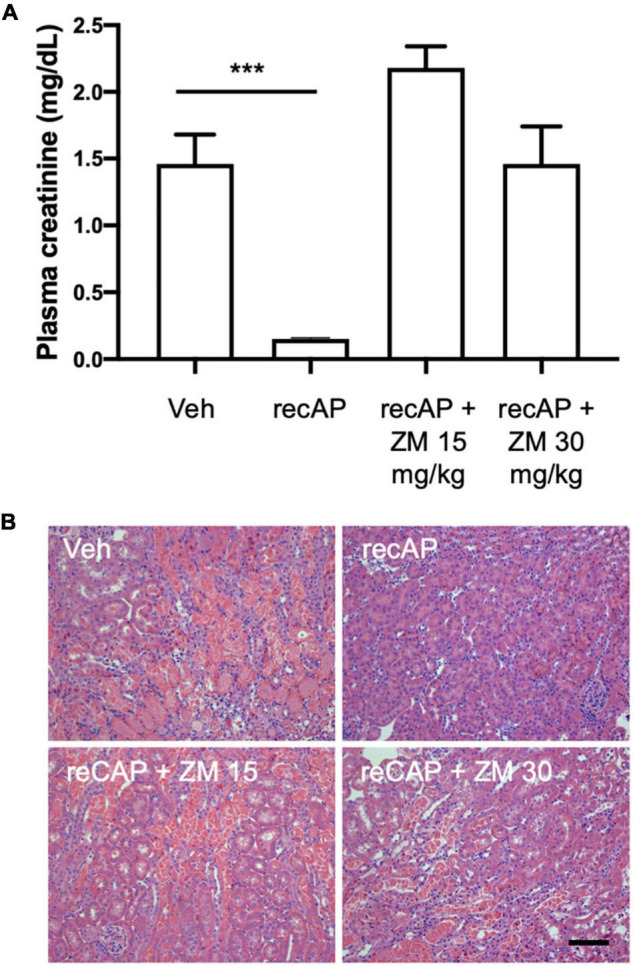
ZM241,385, an adenosine A_2A_ receptor antagonist, reverses the protective effect of ilofotase alfa in male WT mice. WT mice received vehicle (for recAP) or ilofotase alfa (recAP, 2,000 U/kg, i.v.) 1 h before 26 min bilateral kidney ischemia and 24 h of reperfusion. ZM241,385 (ZM) was administered (sc) immediately before ilofotase alfa (recAP, 2,000 U/kg, i.v.). **(A)** Plasma creatinine was measured at 24 h. Plasma creatinine was not significantly different in control groups receiving vehicle for recAP (Veh; 1.46 ± 0.22) vs. vehicle for ZM (1.53 ± 0.06) (not shown in figure). Error bars represent mean ± SEM. **(B)** Representative histology of outer medulla from H&E-stained sections. Scale bar: 100 μm. ***P < 0.001 by one-way ANOVA. Vehicle vs. recAP + ZM. *n* = 4–6.

Alkaline phosphatase is believed to be important for detoxification of a variety of substrates that could participate in tissue injury, however the requirement of enzymatic activity, and perhaps ATP dephosphorylation, for protection led us to propose that adenosine and ARs likely mediate the effect of ilofotase alfa. Stimulation of adenosine 2A receptors (A_2A_R) with an A_2A_R agonist protects kidneys from IRI (32) and requires A_2A_Rs (26), and ZM241,385, an adenosine A_2A_R receptor antagonist, blocks the protective effect of the A_2A_R agonist (32). To investigate the role of A_2A_Rs in ilofotase alfa protection, we administered ZM241,385 at 15 and 30 mg/kg [s.c.; doses selected based on prior experience (25)] immediately prior to the administration of ilofotase alfa, which was given 1 h before IRI (26 min of ischemia and 24 h of reperfusion). Both doses of ZM241,385 completely blocked the protective effect of ilofotase alfa (Figure 3; see also histology, Supplementary Figure 1), consistent with a requirement for A_2A_R-dependent signaling in the mechanism of protection.

Based on our own pharmacokinetic studies of ZM241,385 in C57BL/6 mice, the average exposures of mice to ZM241,385 (at doses of 15 or 30 mg/kg) during the 24 h reperfusion period were above the Ki for A_2A_R but were perhaps too low during the majority of that time period to inhibit A_1_R, A_2B_R, and A_3_R, as judged by the reported K_i_ of ZM241,385 for those AR subtypes (Supplementary Figure 2) (41, 42). Nonetheless, we cannot rule out the possibility that these other AR subtypes are involved in the mechanism of action of ilofotase alfa. Indeed, in *Adora2a^–/–^* mice, which lack A_2A_Rs, ilofotase alfa was less effective than in experiments in WT mice but nevertheless was partially protective (Supplementary Figure 3). Thus, while A_2A_Rs likely contribute to the protection by ilofotase alfa, they may not be completely sufficient for this protection and other mechanisms, including possibly other AR subtypes, may also play a role in the mechanism of action of ilofotase alfa.

### Protection by Ilofotase Alfa Does Not Require Endogenous 5′-Ectonucleotidase

**FIGURE 4:**
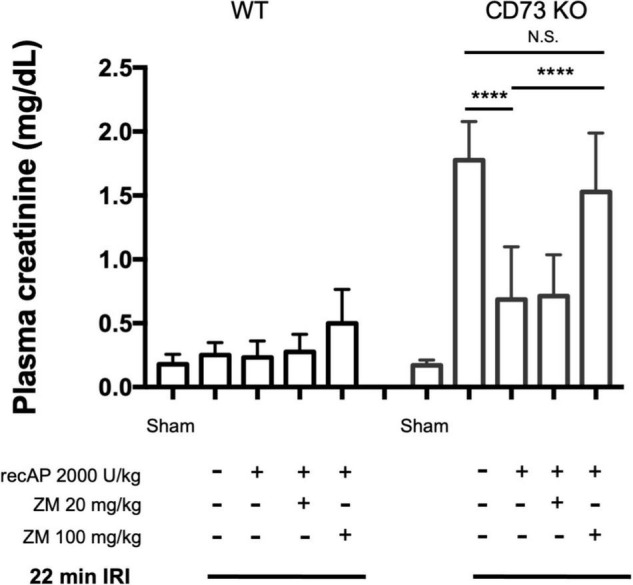
Protective effect of ilofotase alfa does not require the 5′-ectonucleotidase CD73 and is blocked by adenosine receptor antagonist ZM241,385 (ZM). Male *CD73^– /–^* mice (which lack 5′-ectonucleotidase; CD73 KO) are more susceptible to injury than their WT littermate controls and were exposed to 22 min bilateral ischemia, a subthreshold insult that does not produce injury in WT mice. Control mice were subjected to sham surgery. WT and CD73 KO mice received vehicle or ilofotase alfa (recAP, 2,000 U/kg, i.v.) 1 h before 22 min bilateral ischemia and 24 h of reperfusion. Some groups received ZM (20 or 100 mg/kg, sc) immediately before recAP. Plasma creatinine was measured at 24 h. Error bars represent mean ± SEM. Individual data points and additional control groups are shown in Supplementary Figure 4. ****P < 0.0001 by one-way ANOVA. N.S., not significant. *n* = 3–7.

CD39 and CD73 are ectonucleotidases that dephosphorylate ATP to ADP and AMP then AMP to adenosine, respectively. In prior studies we demonstrated that mice lacking CD73, but sufficient in CD39, are more susceptible to IRI than WT mice (25). This enhanced sensitivity is apparent when using a 22 min subthreshold period of ischemia (and 24 h of reperfusion) that does not cause injury in WT mice but induces marked injury in *CD73^–/–^* mice (Figure 4; see also histology, Supplementary Figure 1; individual data points and additional control groups shown in Supplementary Figure 4), as we have shown previously (25). Ilofotase alfa administered 1 h before IRI protected *CD73^–/–^* mice from injury following this mild 22 min of ischemia, which suggests that ilofotase alfa is sufficient in the absence of endogenous CD73 ectonucleotidase activity to generate adenosine by dephosphorylation of ATP, ADP, or AMP, and thereby to provide protection from IRI. This beneficial effect of ilofotase alfa in *CD73^–/–^* mice was blocked by ZM241,385 (Figure 4; dot plots and additional controls in Supplementary Figure 4), consistent with an AR-mediated protective effect.

### Therapeutic Ilofotase Alfa Ameliorates Ischemia-Reperfusion Injury in Mice

**FIGURE 5:**
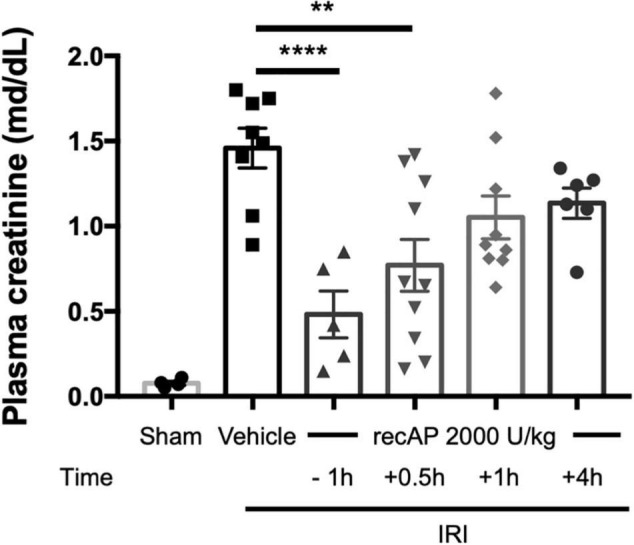
Therapeutic ilofotase alfa protects WT mouse kidneys from injury. Male WT mice received ilofotase alfa (recAP, 2,000 U/kg) 1 h before or 0.5, 1, or 4 h after 26 min bilateral kidney ischemia; kidneys were allowed to reperfuse for 24 h. Control mice were subjected to sham surgery. Plasma creatinine was measured at 24 h. Error bars represent mean ± SEM. **P < 0.01; ****P < 0.001 by one-way ANOVA. *n* = 4–10.

Although ilofotase alfa was protective against kidney IRI when administered prophylactically (Figures 1–4), we sought to determine whether ilofotase alfa could mitigate injury when administered therapeutically to mice, i.e., after the initiation of kidney IRI. Administration of ilofotase alfa 1 h before IRI surgery (using the same parameters as in Figures 1–4, i.e., 26 min of ischemia and 24 h of reperfusion) or 30 min after the ischemic period protected mouse kidneys from IRI, as shown by a statistically significant decrease in plasma creatinine (measured after 24 h of reperfusion) relative to vehicle-treated controls. Smaller degrees of protection were observed when ilofotase alfa administration was delayed by 1 or 4 h after the ischemic period but these reductions in plasma creatinine were not significant (Figure 5). These findings could provide the basis for future studies in a fibrosis model (beyond the scope of the current study) on the ability of ilofotase alfa given after an initial IR insult to prevent progression to fibrosis.

### Therapeutic Ilofotase Alfa Ameliorates Acute Kidney Injury in Rats

To demonstrate efficacy of ilofotase alfa in an additional species and model, the next series of experiments was performed in rats. Initial studies were undertaken to determine the efficacy and dose requirement of ilofotase alfa in a unilateral kidney clamp model of ischemic AKI in male Sprague Dawley rats. A dose of 1,000 U/Kg produced a decrease in plasma creatinine at 24 h after injury, and this dose was used in all subsequent studies in rats. In two subsequent studies in 10–12-week old male Sprague Dawley rats, comparing vehicle to ilofotase alfa (1,000 U/Kg) given immediately after a 30-min period of ischemia, plasma creatinine was significantly reduced 24 h after injury (vehicle vs. ilofotase alfa, 2.62 ± 0.49 vs. 1.45 ± 0.57, *n* = 11, *P* < 0.01) in the ilofotase alpha-treated groups. Ilofotase alfa also increased 24-h urinary creatinine clearance in both studies (vehicle vs. ilofotase alfa, 0.08 ± 0.06 vs. 0.45 ± 0.34 ml/min, *n* = 11, *P* < 0.01). These results showing that ilofotase alfa protected rats in this AKI model are consistent with our findings in mice and provide additional relevance for the therapeutic dosing of ilofotase alfa clinically. This dose of ilofotase alfa (1,000 U/Kg) was then used in the AKI-on-CKD model in which ilofotase alfa treatment was delayed for a longer period of time after ischemic insults.

### Therapeutic Ilofotase Alfa Administration Ameliorates Acute Kidney Injury on a Background of Chronic Kidney Disease

**FIGURE 6:**
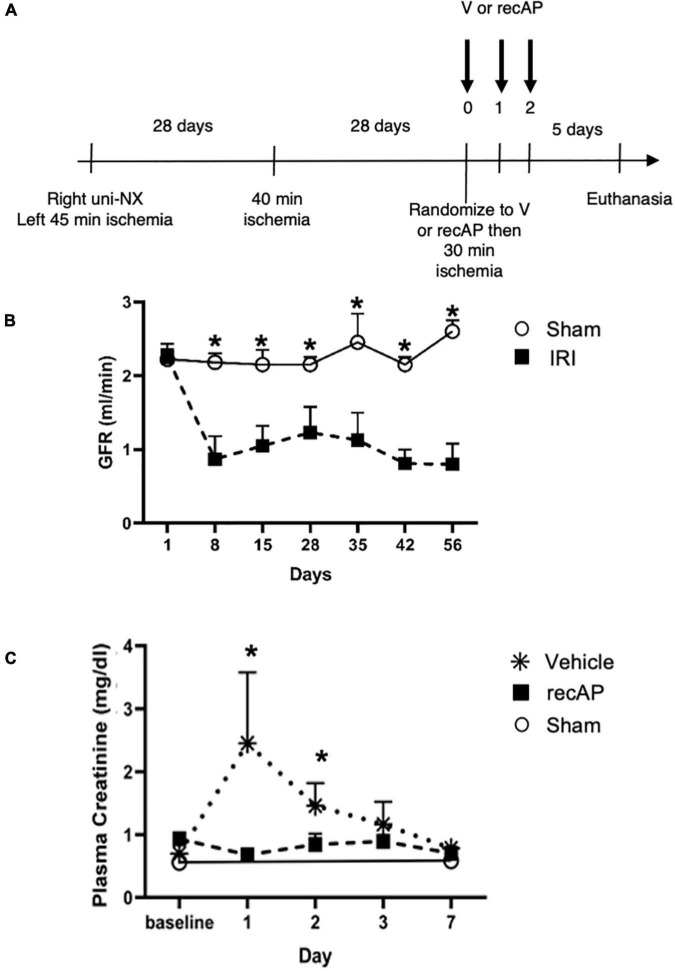
Ilofotase alfa ameliorates kidney injury in rats when administered therapeutically after IRI in the setting of AKI on a background of CKD. **(A)** Timeline for experiments. **(B)** GFR in male rats with unilateral nephrectomy (Uni-NX) and sham unilateral ischemia (open circles) and with Uni-NX and unilateral IRI on days 1 and 28 (filled squares). GFR, calculated from 24-h urinary creatinine clearance. **(C)** Plasma creatinine in CKD rats (after periods of 45, 40, and 30 min of ischemia on days 1, 28, and 56, respectively) at baseline [after randomization to vehicle (V, stars) and recAP (filled squares) groups] and 1, 2, 3, and 7 days later. Open circles, sham controls (Uni-NX and sham unilateral ischemia). *n* = 4 (sham) and 5 (treatment groups). All values are expressed as mean ± SD. *P < 0.01.

In humans, multiple episodes of AKI can often lead to transition to CKD, and realistic animal models are needed for investigating AKI-CKD transition (43). Our AKI studies in rats provided the basis and dosing information for testing ilofotase alfa in a clinically relevant model of AKI to CKD progression. We used a CKD ischemic model in MWF rats involving 3 successive episodes of AKI over 57 days (experimental timeline, Figure 6A) to determine if ilofotase alfa (1,000 U/kg, i.v.) given after the third instance of IRI could significantly reduce kidney injury. A right nephrectomy in the sham ischemic rats had little effect on plasma creatinine, 24-h urinary creatinine clearance (or total GFR, calculated from 24-h urinary creatinine clearance) and proteinuria for the duration of the 8-week study in these aged rats (Table 1, Figure 6B, and Supplementary Figure 5). However, there was a significant increase in plasma creatinine and a decrease in 24-h urinary creatinine clearance in the rats undergoing right nephrectomy and left IRI. There was also a significant increase in urinary protein following the first clamp, and all of these changes persisted throughout the 8-week study, clearly indicative of a state of CKD.

**TABLE 1:** Plasma creatinine, GFR, and urinary protein in rats with unilateral (right) nephrectomy and sham unilateral ischemia and with unilateral nephrectomy and unilateral IRI on days 1 and 28.

	Baseline Day 1	1 week after 1st clamp Day 8	2 week after 1st clamp Day 15	2nd baseline Day 28	1 week after 2nd clamp Day 35	2 weeks after 2nd clamp Day 42
**Plasma creatinine (mg/dl)**						
Nephrectomy	0.47 ± 0.04	0.49 ± 0.02	0.39 ± 0.05	0.51 ± 0.01	0.49 ± 0.04	0.52 ± 0.04
Nx + Clamp	0.47 ± 0.03	0.84 ± 0.09	0.66 ± 0.06	0.75 ± 0.13	0.75 ± 0.21	0.90 ± 0.19
*P*-value	NS	<0.01	<0.01	<0.01	<0.01	<0.01
**GFR (ml/min/100 g body weight)**						
Nephrectomy	0.73 ± 0.04	0.69 ± 0.01	0.66 ± 0.02	0.63 ± 0.03	0.69 ± 0.12	0.65 ± 0.08
Nx + Clamp	0.73 ± 0.03	0.29 ± 0.11	0.34 ± 0.08	0.35 ± 0.10	0.32 ± 0.11	0.22 ± 0.06
*P*-value	NS	<0.01	<0.01	<0.01	<0.01	<0.01
**Urinary protein (mg/24 h/GFR)**						
Nephrectomy	19.7 ± 6.8	18.3 ± 3.5	18.5 ± 3.0	22.7 ± 3.9	23.7 ± 2.9	28.1 ± 3.0
Nx + Clamp	24.3 ± 6.5	69.4 ± 33.7	53.6 ± 22.4	65 ± 38.8	54.0 ± 29.3	80.9 ± 37.8
*P*-value	NS	<0.01	0.01	0.01	<0.01	<0.01

*Sham rats (n = 6) underwent a right nephrectomy (Nx) on day 1 and subsequent sham IRI surgeries on day 1 and 28. Chronic kidney disease rats (Nx + Clamp) (n = 14 initially, 12 at end of study) underwent right nephrectomy and 45-min clamp on left kidney on day 1. On day 28 CKD rats underwent a 40-min clamp. GFR calculated from 24-h urinary creatinine clearance and corrected for body weight. Values are mean ± SD. Statistical significance at each time point was determined by a one-sided Student’s t-test.*

At randomization two weeks after the second clamping procedure, the IRI rats had a plasma creatinine and 24-h urinary creatinine clearance of 0.90 ± 0.19 mg/dl and 0.22 ± 0.06 ml/min/100 g body weight compared to nephrectomized only sham rats with a plasma creatinine and 24-h creatinine clearance of 0.52 ± 0.04 and 0.65 ± 0.08, respectively. They also had increased proteinuria of 80.9 ± 37.8 vs. 28.1 ± 3.0 mg/24 h/GFR, respectively. Figure 6C shows the effect of ilofotase alfa treatment when the CKD rats underwent a third clamp, this time for 30 min (vehicle or ilofotase alfa administered at 0, 24 and 48 h after clamp release). On days 1 and 2 following IRI, ilofotase alfa minimized ischemic injury as measured by plasma creatinine, which was most elevated from baseline in vehicle-treated groups on days 1 and 2. On subsequent days, creatinine continued to decline, nearly reaching baseline on day 7, at which point ilofotase alfa had no additional effect. Measures of 24-h urinary creatinine clearances on day 7 after the third clamp (0.40 ± 0.15 vs. 0.25 ± 0.09 ml/min/100 g body weight, *P* < 0.05) showed that this difference persisted. Twenty four-hour urinary protein was not statistically different at 7 days following injury between the two groups (data not shown).

## Discussion and Conclusion

**FIGURE 7:**
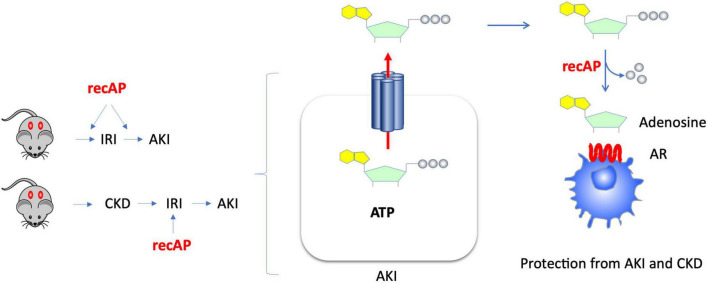
Kidney protection from AKI or CKD with prophylactic or therapeutic administration of ilofotase alfa. AKI causes release of ATP from damaged or dying cells. Administration of ilofotase alfa (human recombinant alkaline phosphatase, recAP), either before or after ischemia-reperfusion injury (IRI, a model of AKI) and after ischemic events in an AKI-on-CKD model, prevented or reduced kidney injury and preserved kidney function. Extracellular ATP can be fully dephosphorylated by ilofotase alfa (recAP) to produce adenosine. The projective effect of ilofotase alfa is likely mediated by adenosine receptor (AR) signaling.

The use of recombinant alkaline phosphatase (recAP) in preclinical and clinical studies for the treatment of sepsis-associated AKI and for reduction of mortality in sepsis has yielded promising results. Alkaline phosphatase (AP) is an endogenous enzyme that is expressed ubiquitously and that is responsible for dephosphorylating a variety of molecules. Several mechanisms have been proposed to account for the efficacy of ilofotase alfa in preventing sepsis-associated AKI when administered to animals. We now show that ilofotase alfa, a human recombinant AP, which is being tested clinically, is also efficacious in preventing kidney injury in IRI models of AKI in both mice and rats, and furthermore we show that it is efficacious when used therapeutically, i.e., after IRI, to reduce injury in rodent AKI models and in an AKI-on-CKD model in rats. Our data strongly suggest that the ability of ilofotase alfa to sequentially dephosphorylate ATP to produce adenosine (16, 19, 24) leads to AR signaling partly *via* A_2A_ receptors to produce protective effects that are known to reduce AKI (Figure 7); removal of extracellular ATP in this manner could also prevent ATP’s deleterious effects through its actions as a DAMP mediated by P2 receptors (not shown in figure). Prior studies have examined the effect of recAP or other preparations of AP on sepsis-associated AKI, however our study examined the protective effects of ilofotase alfa in AKI and CKD in rodents (rats and mice) without the widespread inflammation that occurs in sepsis.

### Ilofotase Alfa-Induced Mechanisms of Protection

During ischemic injury and other tissue injury processes, ATP is released from damaged and dying cells (23). Extracellular ATP is a substrate for AP (16). ATP can be dephosphorylated sequentially by phosphatases to produce adenosine, and AP (as well as other nucleotidases and phosphatases) can metabolize AMP to adenosine (44, 45). As APs as a class of enzymes can remove all three phosphate groups from ATP (16, 19, 24) to yield adenosine, ilofotase alfa can potentially shift the tissue environment and that of circulating immune cells from pro-inflammatory to anti-inflammatory in a context-dependent fashion (i.e., if there is no extracellular ATP or ADP, then recAP has no effect). The importance of a shift in substrates for the purinergic pathways in acute and CKD through ATP dephosphorylation and the potential for targeting these pathways for the development of novel drugs has been reviewed recently (46).

Adenosine protects tissues from ischemic injury and exerts anti-inflammatory effects, and kidneys are protected from IRI by activation of A_2A_Rs (including activation of A_2A_Rs on immune cells) (26–34) or A_2B_Rs (47). Our current studies are consistent with the hypothesis that adenosine generated by ilofotase alfa binds to A_2A_Rs and contributes to protection by recAP, as supported by the finding that ZM241,385, a selective antagonist of A_2A_Rs, blocks the protection. However, protection can occur through adenosine binding to either A_2A_Rs or A_2B_Rs (which are both Gs-coupled receptors) resulting in activation of adenylate cyclase, elevation of cAMP, and subsequent stimulation of protein kinase A. The involvement of each receptor subtype will also be dependent on the local concentration of ATP during injury and that of adenosine generated by ilofotase alfa. At lower levels of ATP and adenosine, the effect on A_2A_Rs would predominate, whereas higher levels of adenosine would engage A_2B_Rs. Although the protective effects of ilofotase alfa were blocked completely by ZM241,385 in WT mice, they were only partially blocked by the absence of A_2A_Rs in *Adora2a^–/–^* mice, suggesting the possibility that other AR subtypes and/or other mechanisms are also involved. Both A_2A_Rs and A_2B_Rs have excess capacity to provide protection, and stimulation of either receptor (or both) could give full protection. In *CD73^–/–^* mice, complete inhibition of the protective effects of recAP was found only at higher doses of ZM241,385 (100 mg/kg), which could be blocking A_2A_Rs and A_2B_Rs, and more transiently A_1_Rs, as supported by our pharmacokinetic studies (Supplementary Figure 2). Administration of 22.3 and 92.3 mg/kg ZM241,385 (nominally 20 and 100 mg/kg) yielded free plasma concentrations immediately after dosing of 366 and 2,095 nM, respectively. Plasma levels of ZM241,385 for the duration of the 24 h following both of these doses remained substantially greater than the K_i_ for mouse A_2A_Rs and at early, but not later, time points were above the K_i_ for mouse A_1_R and A_2B_R. Given the experimental timeline used in the IRI studies (i.e., ZM241,385 and ilofotase alfa administered sequentially 1 h before IRI and mice euthanized 24 h later), it is possible that ZM241,385 blocked A_2A_Rs, A_2B_Rs and perhaps A_1_Rs at some point during the course of the experiment.

Ilofotase alfa partially protected *Adora2a^–/–^* mice from kidney IRI, compared to WT mice, again indicating a role for A_2A_Rs, other ARs and potentially other effectors in the protective mechanism of ilofotase alfa. A_2A_Rs do not appear to be necessary for recAP’s ability to reduce LPS-induced inflammation *in vitro* (recAP was able to attenuate inflammation in proximal tubule cells isolated from *Adora2a^–/–^* mice) (21) but this is a different model than kidney IRI and protection *in vivo* requires activation of A_2A_Rs on immune cells but not on tubule cells (26). Still, these *in vitro* experiments suggest an additional protective mechanism for recAP, one with direct effects on tubule cells, that does not involve A_2A_Rs.

In addition to reducing injury through AR-mediated effects, ilofotase alfa may also protect kidneys by metabolizing ATP, reducing levels of extracellular ATP, and thereby reducing the deleterious effects resulting from ATP’s action as a DAMP. Similarly, recAP provides protection from sepsis by dephosphorylating and detoxifying LPS (7, 37), and because ZM241,385 did not block protection by recAP, the authors concluded that recAP protected kidneys in their model primarily by removal of ATP and ADP by dephosphorylation (37). recAP and other forms of AP can dephosphorylate both LPS and ATP (12, 13, 16).

### Ilofotase Alfa Administration to Mice Deficient in 5′-Ectonucleotidase (CD73)

The ectonucleotidases CD39 and CD73 sequentially dephosphorylate ATP to ADP and AMP (CD39) and AMP to adenosine (CD73). These ectonucleotidases and endogenous AP in the kidney vasculature can metabolize AMP to adenosine (45). In the current studies, kidney injury was reduced in *CD73^–/–^* mice by prior administration of ilofotase alfa, and the protection was inhibited by ZM241,385 at the highest dose. These results suggest that ilofotase alfa is sufficient to rescue the deficit in adenosine-mediated protective effects, due to CD73 deficiency, that contribute to increased kidney injury in *CD73^–/–^* mice (25) and that the protective effect is mediated by AR signaling. The degree of protection produced by ilofotase alfa in *CD73^–/–^* mice was not as great as in WT mice. The ability of ilofotase alfa to dephosphorylate AMP to adenosine, which is not needed in WT mice that have functional 5′-ectonucleotidase activity, is likely necessary for the protective effect in *CD73^–/–^* mice.

### Efficacy of Ilofotase Alfa in an Acute Kidney Injury-on-Chronic Kidney Disease Model in Rats

The lack of translation of successful therapies in preclinical AKI models has been a challenge in advancement of therapy for clinical AKI. However, IRI studies to date have been accomplished in model systems that utilize young and otherwise normal rodents, and the clinical studies are conducted in patients with a high risk of AKI, most of whom have CKD and are proteinuric. Therefore, we used our model of aged rats with CKD and proteinuria based on recurrent episodes of IR-induced AKI to test whether the effect of ilofotase alfa would still be present in rats with significant comorbidities including a reduced GFR. We used a right nephrectomy and left unilateral kidney pedicle clamp model in aged, proteinuric MWF rats to induce CKD. This rat strain is known to have a reduced number of glomeruli and develop progressive spontaneous proteinuria at 12 weeks in males and 16 weeks in females (48). A month was allowed between two episodes of IRI to allow for full recovery from the previous injury. As expected, the right nephrectomy alone resulted in virtually no change in either plasma creatinine or GFR, again demonstrating the capacity for renal reserve to mask large reductions in function even in older rodents. In this model ilofotase alfa administration was increased to three total doses, one at the time of clamp removal and additional doses at 24 and 48 h after injury. This approach proved highly effective in minimizing plasma creatinine on days 1 and 2 and urinary creatinine clearance when it was measured on day 7 and indicates a clear therapeutic efficacy for recAP when administered after an acute episode of ischemia precipitated after a protracted period of CKD. In future studies important clinically relevant information could be gained by testing whether administration of ilofotase alfa in earlier stages after IRI prevents development of CKD. Although therapeutic efficacy has so far been demonstrated only when ilofotase alfa was given 30 min prior to or immediately after IRI in our AKI models in mice and rats, respectively, it is possible that ilofotase alfa could have clinical utility in humans to prevent AKI in elective surgery cases. Additionally, because onset of AKI often cannot be predicted in humans, it will be important to determine if multiple doses of recAP could be more effective than a single dose in established AKI.

The doses found to be effective in our rodent models are highly relevant from a therapeutic perspective in patients. The dose of 1,000 U/Kg was the highest dose used in the STOP-AKI trial that resulted in statistically significant improvement of kidney function at 21 and 28 days, as measured by improvement of endogenous creatinine clearance. In the same study, the dose of 1,000 U/Kg also improved survival of the patients compared to placebo. The dose of 1,000 U/Kg is currently being tested in the Phase 3 clinical trial REVIVAL (NCT04411472) for sepsis-associated AKI.

In summary, we have demonstrated that ilofotase alfa is effective in rodents in preventing and treating both AKI and AKI-on-CKD, and our results suggest a mechanism that is dependent on adenosine-mediated tissue protective effects, which are dependent at least in part on A_2A_Rs. Other AR subtypes may contribute, including A_2B_Rs and A_1_Rs. These mechanistic studies are supportive of clinical trials of ilofotase alfa for sepsis-associated AKI (15, 17, 49) that are currently being conducted in a global Phase 3 pivotal study, and they support the therapeutic potential of ilofotase alfa as a treatment option for AKI.

## Data Availability Statement

The original contributions presented in the study are included in the article/Supplementary Material, further inquiries can be directed to the corresponding author.

## Ethics Statement

The animal study was reviewed and approved by the University of Virginia Animal Care and Use Committee and the Indiana University IACUC.

## Author Contributions

DR, JH, NK, JG, BM, AVE, and MDO designed the experiments. LH, SZ, DR, AB, KB, EM, JL, GH, IE, RS, and SC-B conducted the experiments and acquired and analyzed the data. DR, BM, JH, AVE, and MDO wrote and edited the manuscript. All authors approved the final version of the manuscript.

## Author Disclaimer

The content is solely the responsibility of the authors and does not necessarily represent the official views of the National Institutes of Health. With the exception of the Conflicts of Interest Statement, all authors declare no other competing interests.

## Conflict of Interest

JH, AB, KB, EM, JL, GH, NK and JG were employed by Pfizer Inc. AVE was employed by AM-Pharma B.V. MDO had a research grant from Pfizer Inc. AVE was consultant to AM-Pharma B.V. and a named inventor on patent filings related to ilofotase alfa. BM had research grants with AM-Pharma B.V. and Pfizer Inc. and was on a MAB for AM-Pharma B.V. The remaining authors declare that the research was conducted in the absence of any commercial or financial relationships that could be construed as a potential conflict of interest.

## Publisher’s Note

All claims expressed in this article are solely those of the authors and do not necessarily represent those of their affiliated organizations, or those of the publisher, the editors and the reviewers. Any product that may be evaluated in this article, or claim that may be made by its manufacturer, is not guaranteed or endorsed by the publisher.

## Abbreviations

A_2A_R: adenosine A_2A_ receptor
AKI: acute kidney injury
AP: alkaline phosphatase
CKD: chronic kidney disease
H&E: hematoxylin and eosin
IRI: ischemia-reperfusion injury
Mut-recAP: mutant recap
recAP: recombinant alkaline phosphatase.
