# Supplementary material for: Human Recombinant Alkaline Phosphatase (Ilofotase Alfa) Protects Against Kidney Ischemia-Reperfusion Injury in Mice and Rats Through Adenosine Receptors
Source: Front Med (Lausanne). 2022 Jul 28;9:931293. doi: 10.3389/fmed.2022.931293 (PMC9366018; doi:10.3389/fmed.2022.931293)
Supplement: Supplementary file 1 [file Data_Sheet_1.PDF]

Supplemental Figures
Table of Contents

Supplemental Figure 1: Representative histology of H&E-stained sections for main Figures 2-4

Supplemental Figure 2: Pharmacokinetics of adenosine receptor antagonist ZM241,385 in C57BL/6 female mice

Supplemental Figure 3: Contribution of adenosine A2A receptors to the protective effect of ilofatase alfa in mice

Supplemental Figure 4: Protective effect of ilofatase alfa does not require the 5'-ectonucleotidase CD73 and is blocked by adenosine receptor antagonist ZM241385 (ZM)

Supplemental Figure 5: Plasma creatinine in AKI on CKD model in rats prior to randomizing for ilofatase alfa treatment

Supplemental Figure 1: Representative histology of H&E-stained sections for main Figures 2-4 demonstrates for main treatment groups that marked changes in creatinine are supported by marked changes in kidney histology

In each set of figures for Supplemental Figure 2, the top panel is a stitched image of the entire kidney. Using an MBF Bioscience and Zeiss AxioImager Z1 microscope system, virtual tissue section images were acquired using StereoInvestigator software (version 11, MBF Bioscience, Williston, VT). Parameters were defined in this application of the software so that images of kidney sections acquired with a 5X objective were automatically stitched together to produce a full view of the kidney section that can be magnified digitally from the image with preservation of the original optical resolution.

Magnified images of the same tissue section are shown in the middle and bottom panels of each set of figures. Area depicted in middle panels includes cortex (from edge of tissue section) through outer medulla and in some cases outer edge of inner medulla. Bottom panels are primarily outer medulla. Example: boxes in first set of images show area of kidney section that was photographed at higher magnification.

Scale bar: 400 μm (top panel), 200 μm (middle panel), 100 μm (bottom panel)

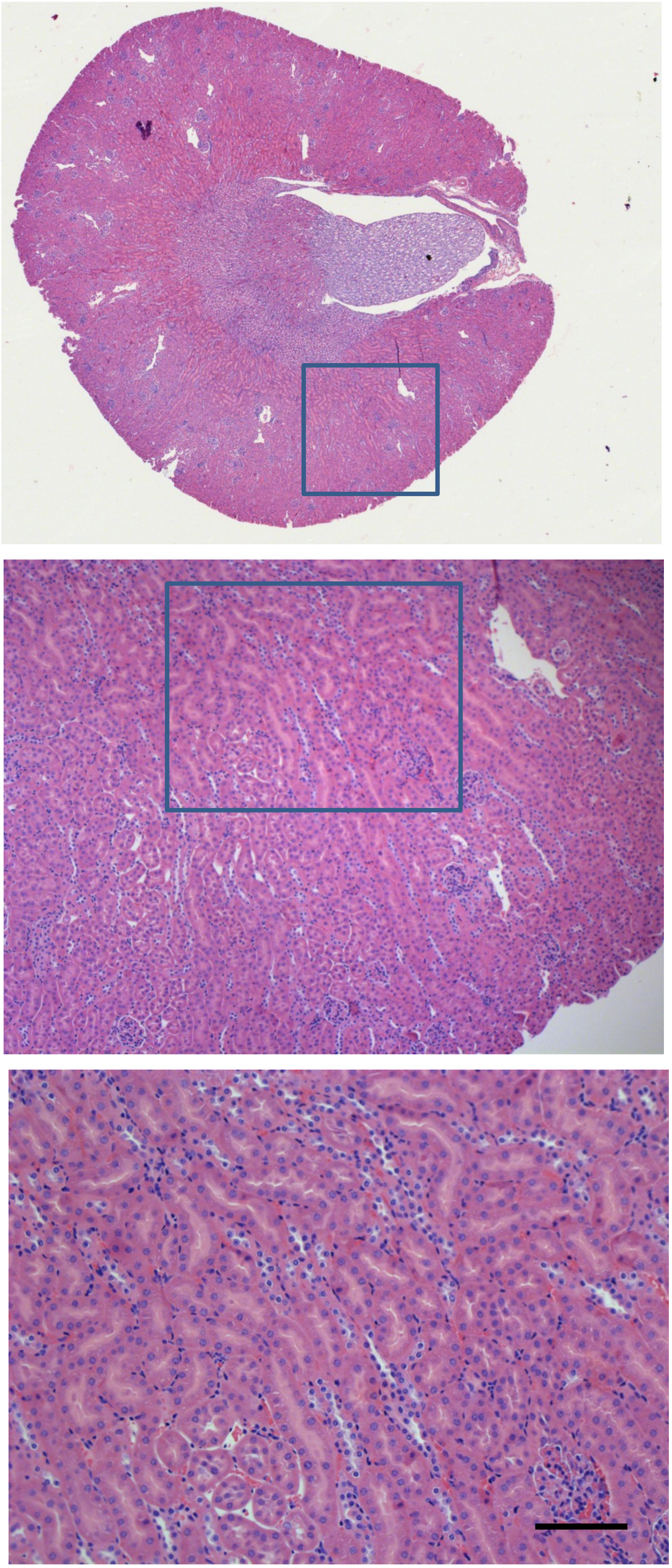

Representative histology of H&E-stained sections for main Figure 2: sham

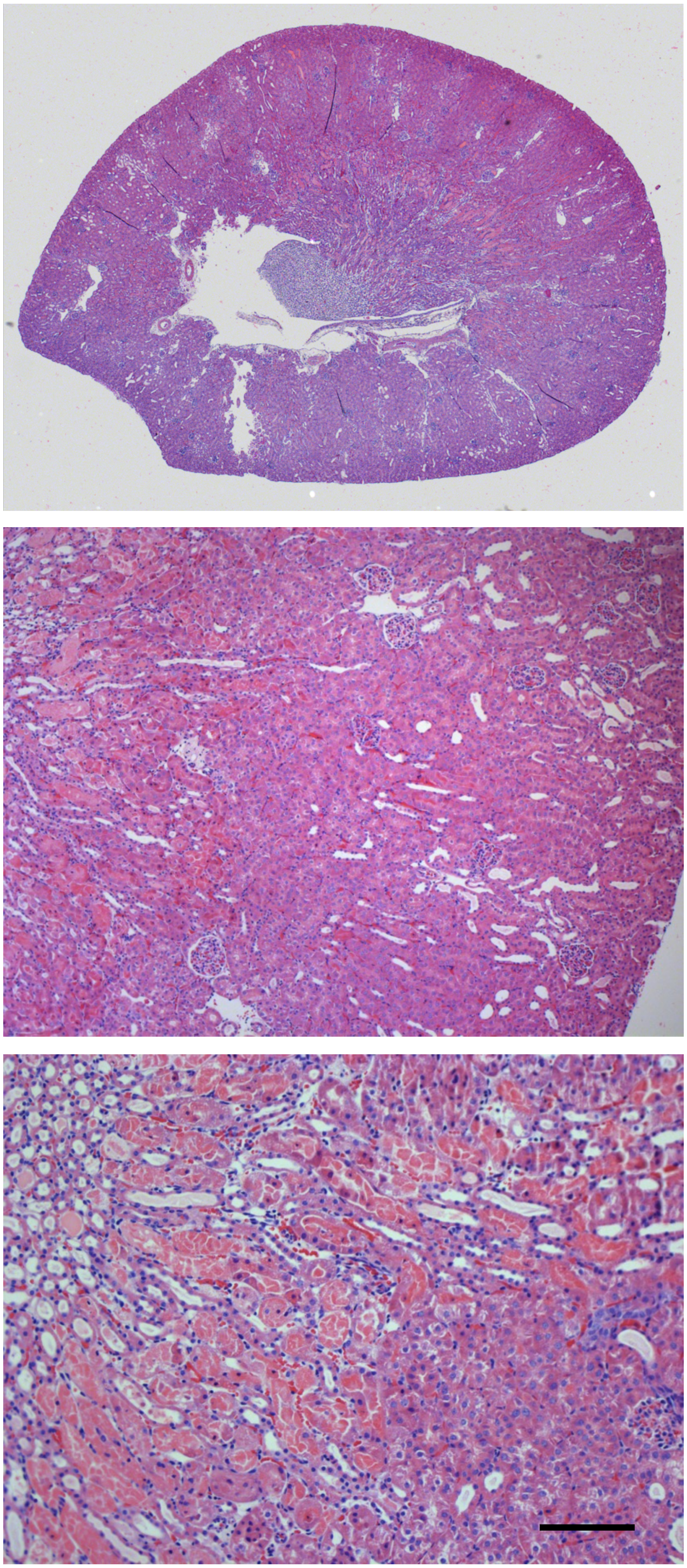

Representative histology of H&E-stained sections for main Figure 2: vehicle

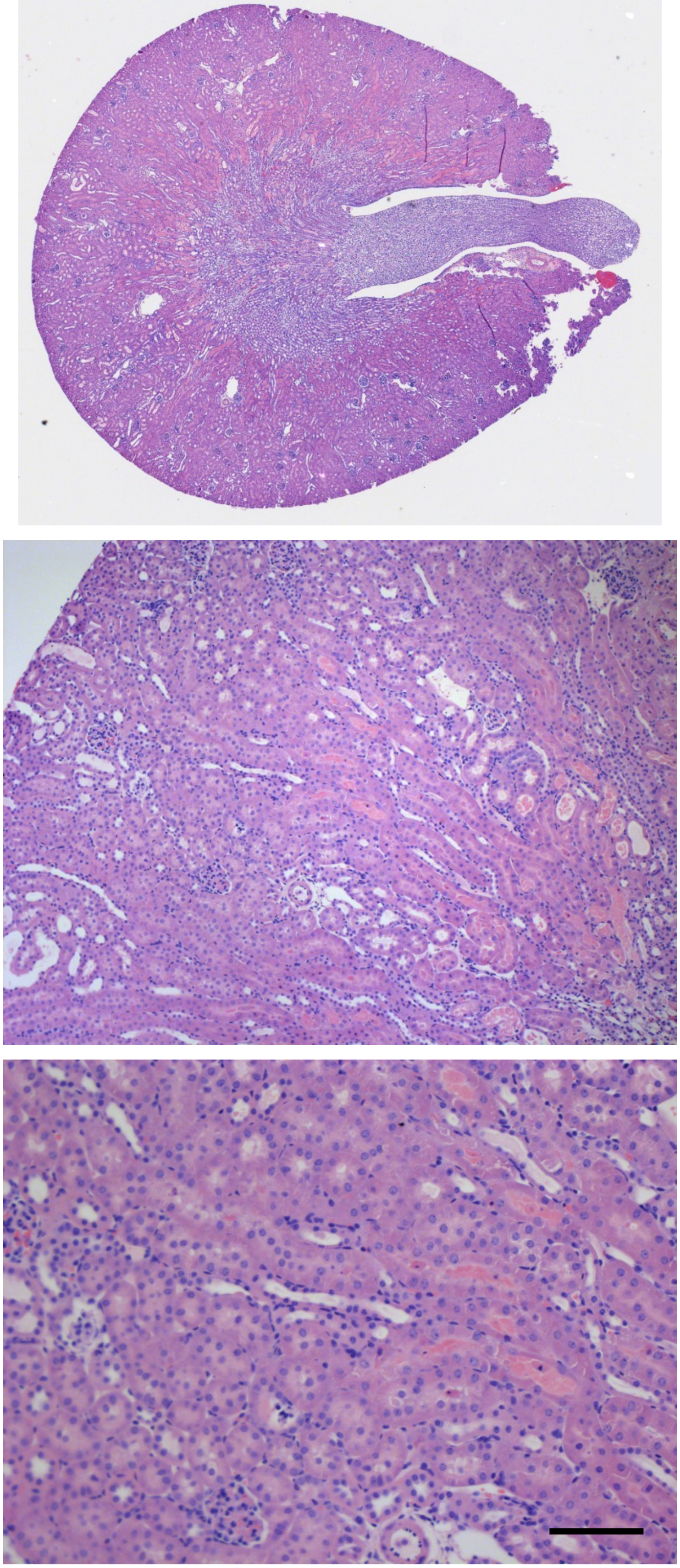

Representative histology of H&E-stained sections for main Figure 2: recAP

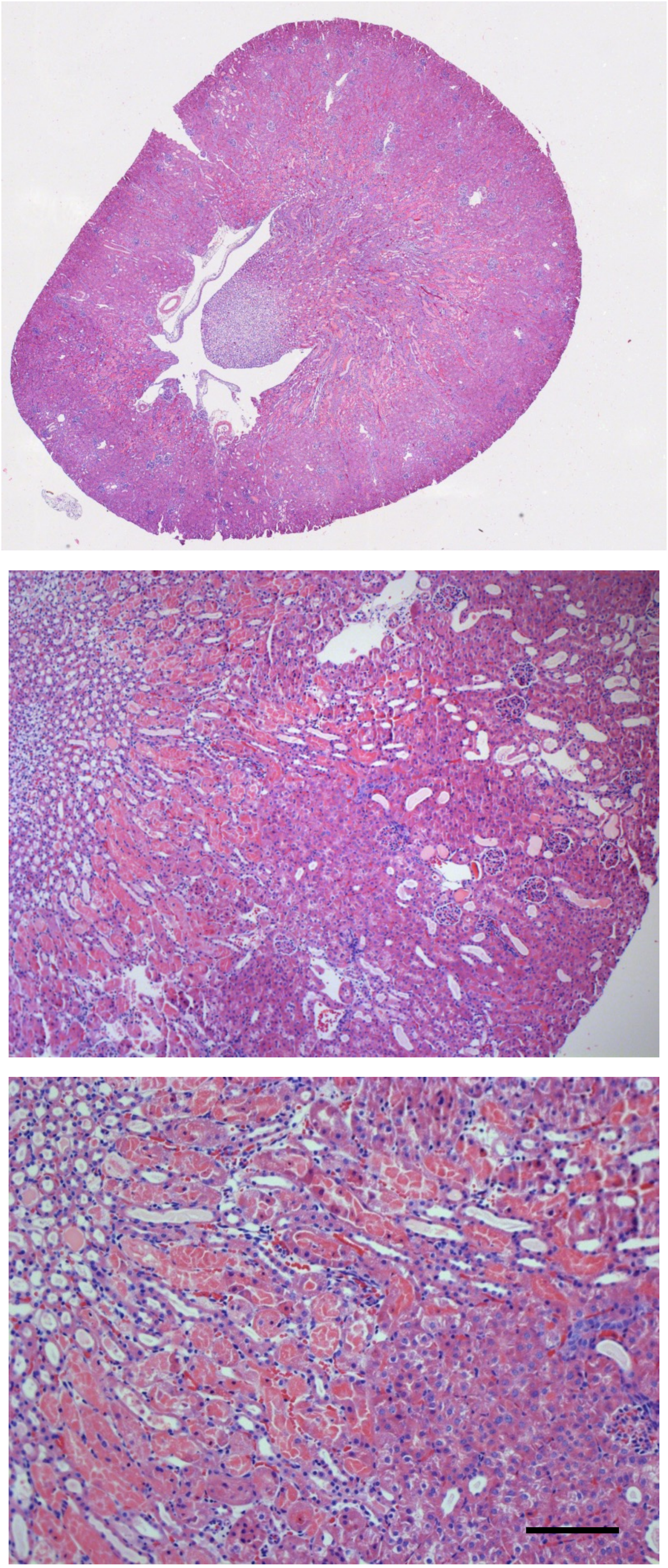

Representative histology of H&E-stained sections for main Figure 2:
mutant recAP

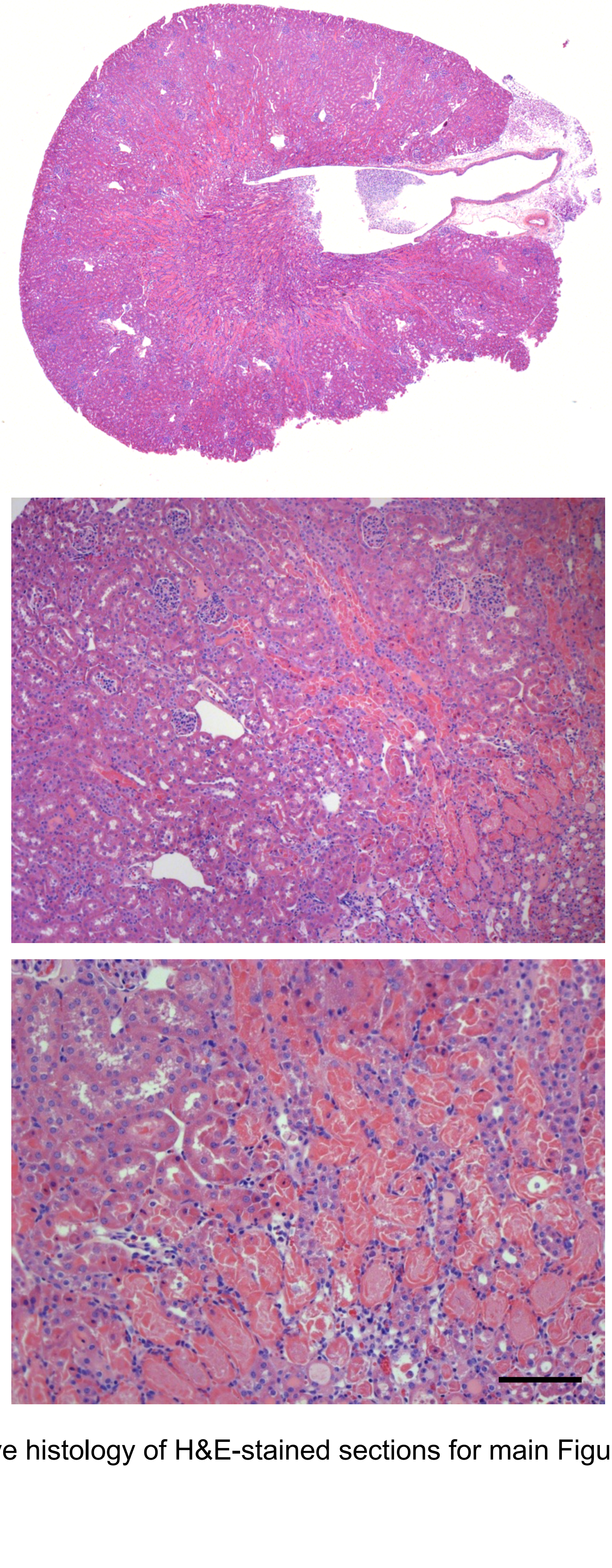

Representative histology of H&E-stained sections for main Figure 3: vehicle

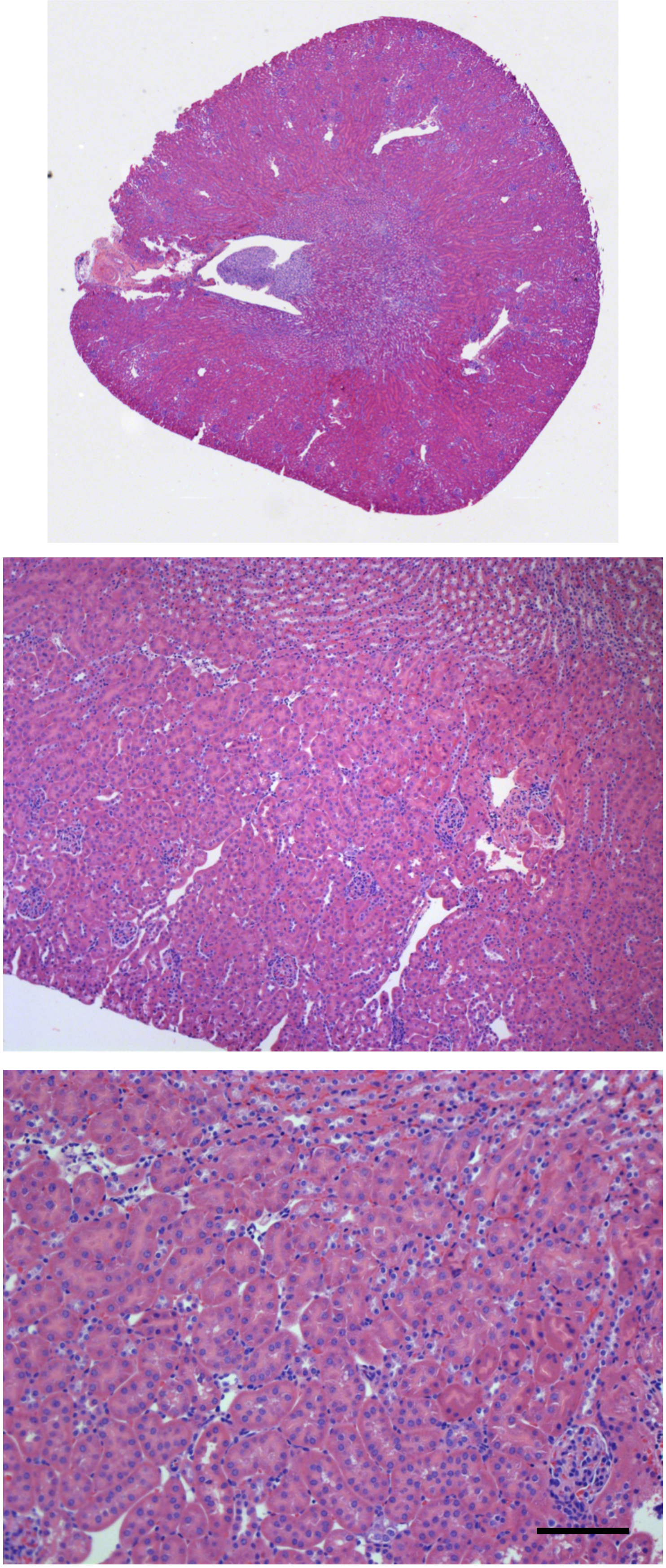

Representative histology of H&E-stained sections for main Figure 3: recAP

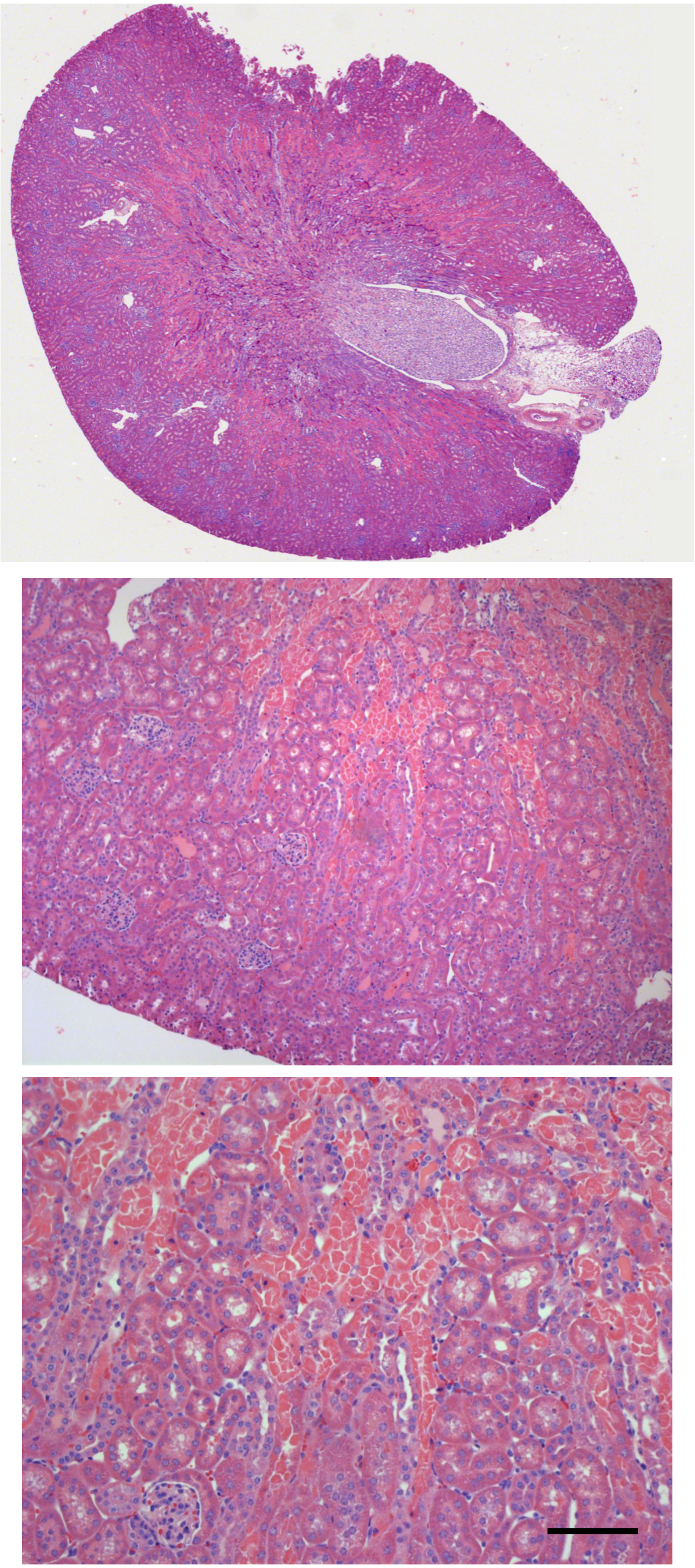

Representative histology of H&E-stained sections for main Figure 3:
recAP + 15 mg/ml ZM241,385

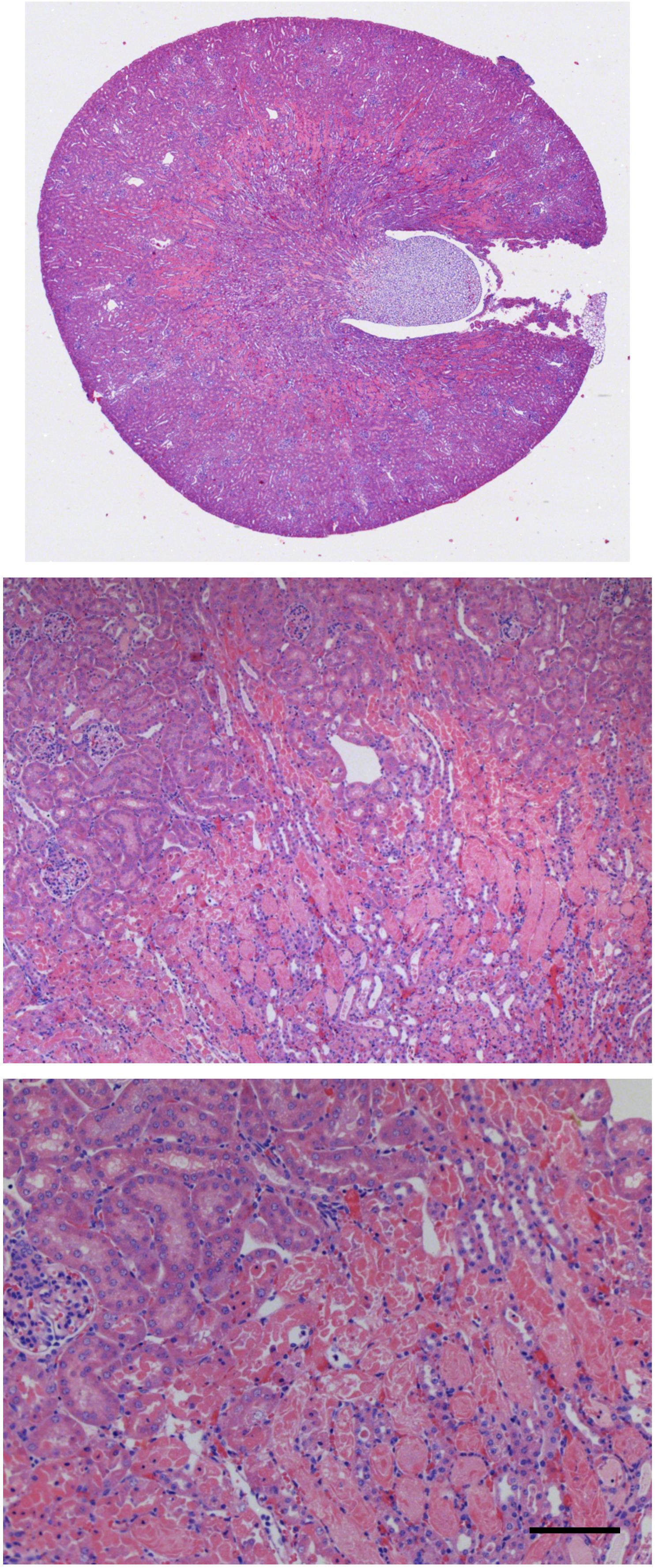

Representative histology of H&E-stained sections for main Figure 4:
vehicle in CD73 KO

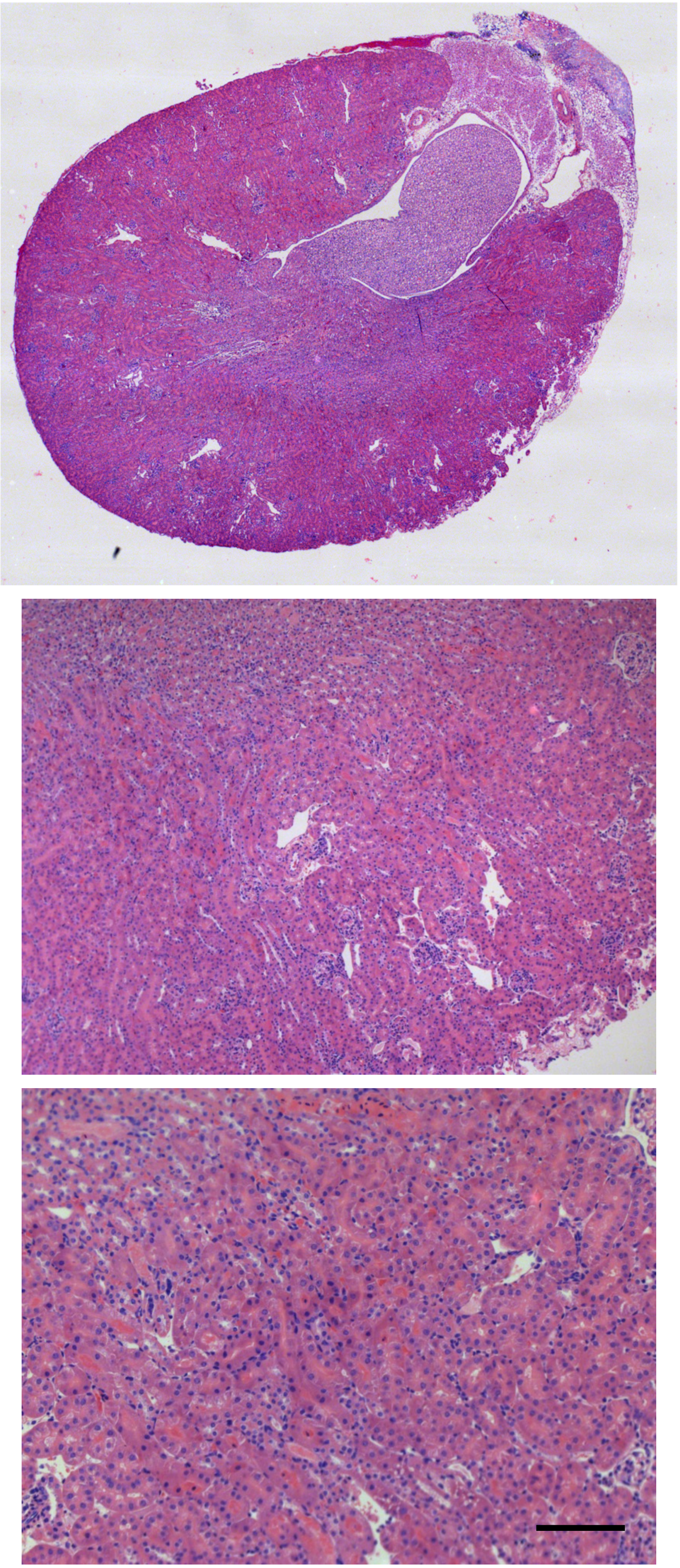

Representative histology of H&E-stained sections for main Figure 4: recAP in CD73 KO

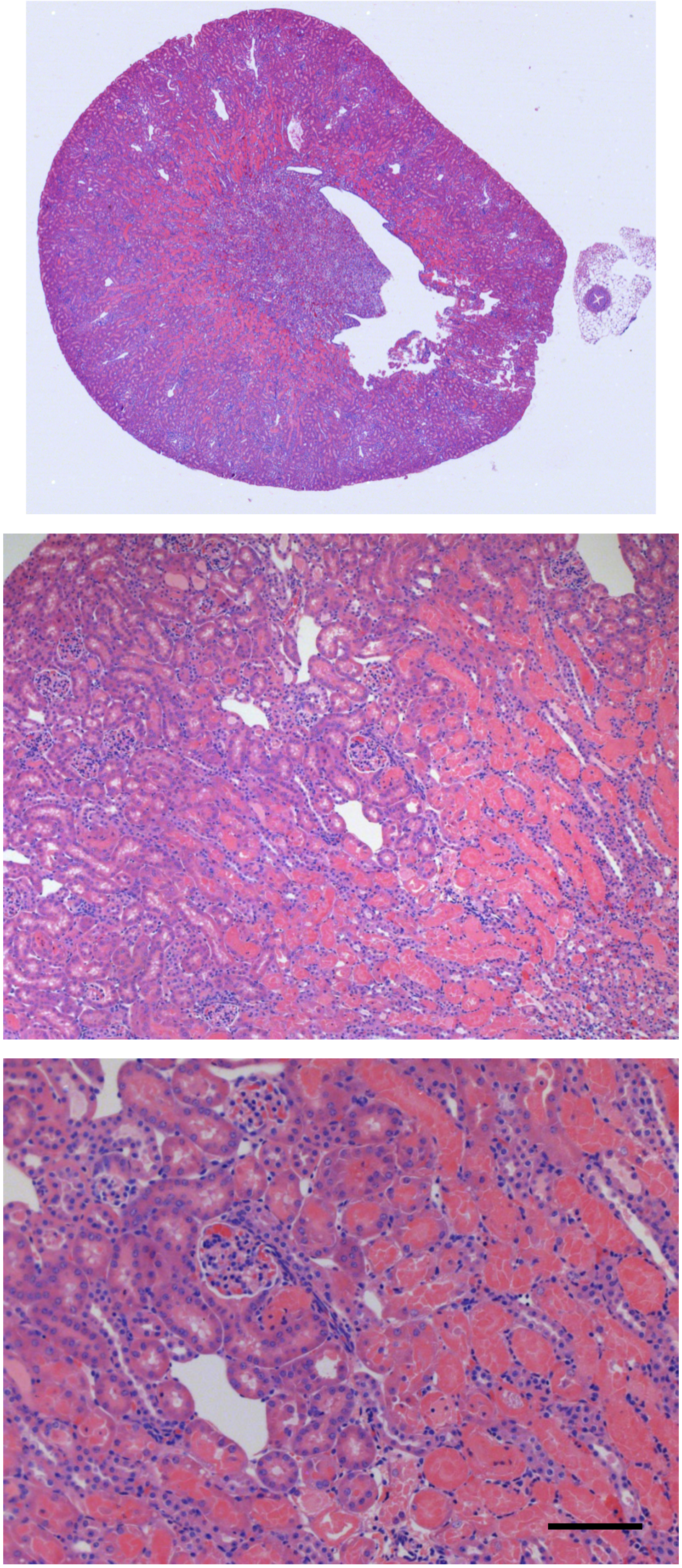

Representative histology of H&E-stained sections for main Figure 4:
recAP + 100 mg/kg ZM241,385 in CD73 KO

Supplemental Figure 2. Pharmacokinetics of adenosine receptor antagonist ZM241,385 in C57BL/6 female mice

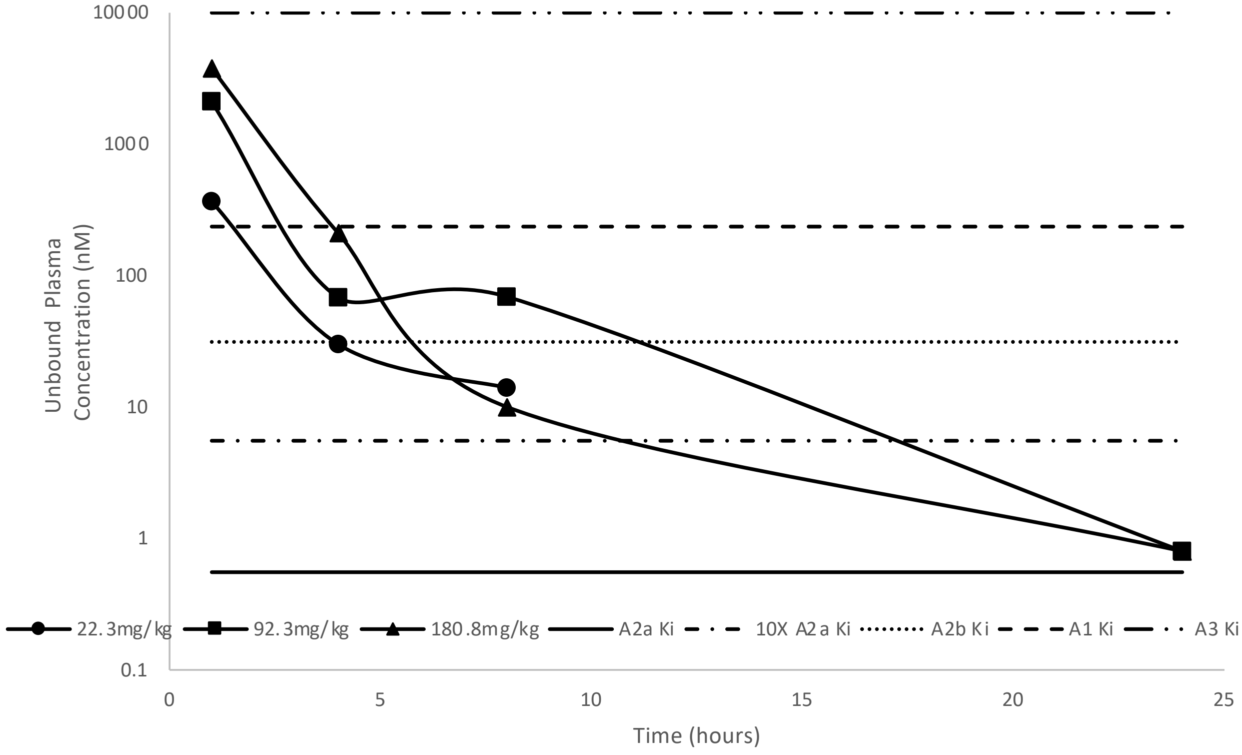

ZM241,385 was administered (single dose of 22.3, 92.3 or 180.8 mg/kg; s.c.) to C57BL/6 female mice (~6-8 weeks old; n= 3/group). Mice were euthanized 1, 4, 8 and 24 hours later, and blood was collected by cardiac puncture. Plasma concentration of ZM241,385 was determined by liquid chromatography–tandem mass spectrometry (LC-MS/MS). The table of published K_i values for ZM241,385 at each of the adenosine receptors subtypes is provided for reference.

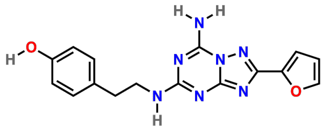

ZM241,385

Published K_i values for ZM241,385[1,2]

**ZM241,385**	**Ki nM**		**Ki nM**		**Ki nM**
**Human A1**	**774**	**Rat A1**	**684**	**Mouse A1**	**236**
**Human A2A**	**1.6**	**Rat A2A**	**1.25**	**Mouse A2A**	**0.554**
**Human A2B**	**75**	**Rat A2B**	**373**	**Mouse A2B**	**31.3**
**Human A3**	**743**	**Rat A3**	**>10000**	**Mouse A3**	**>10000**

1. Fredholm, BB, AP, IJ, Jacobson, KA, Linden, J, Muller, CE: International Union of Basic and Clinical Pharmacology. LXXXI. Nomenclature and classification of adenosine receptors--an update. *Pharmacol Rev*, 63: 1-34, 2011.
2. Alnouri, MW, Jepards, S, Casari, A, Schiedel, AC, Hinz, S, Muller, CE: Selectivity is species-dependent: Characterization of standard agonists and antagonists at human, rat, and mouse adenosine receptors. *Purinergic Signal*, 11: 389-407, 2015

Supplemental Figure 3. Contribution of adenosine A2A receptors to the protective effect of recAP in mice

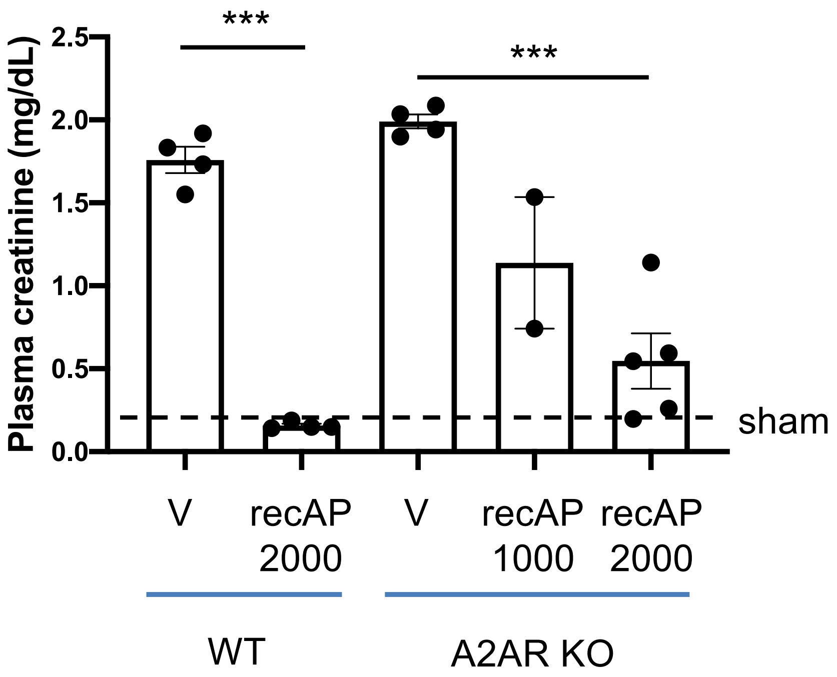

Adenosine A2A receptors contribute to the protective effect of recAP in mice. Vehicle (V) or recAP was administered (i.v.) to WT or *Adora2a*$^{-/-}$ (adenosine A2A receptor KO, A2A KO) mice 1 hr before 26 min bilateral kidney ischemia and 24 hr of reperfusion. Plasma creatinine was measured at 24 hr. Horizontal dashed line represents level of plasma creatinine in control mice exposed to sham surgery. Error bars represent mean ± SEM. ***, $P < 0.0001$ by one-way ANOVA. n = 2-5.

Supplemental Figure 4. Protective effect of recAP does not require the 5'-ectonucleotidase CD73 and is blocked by adenosine receptor antagonist ZM241385 (ZM)

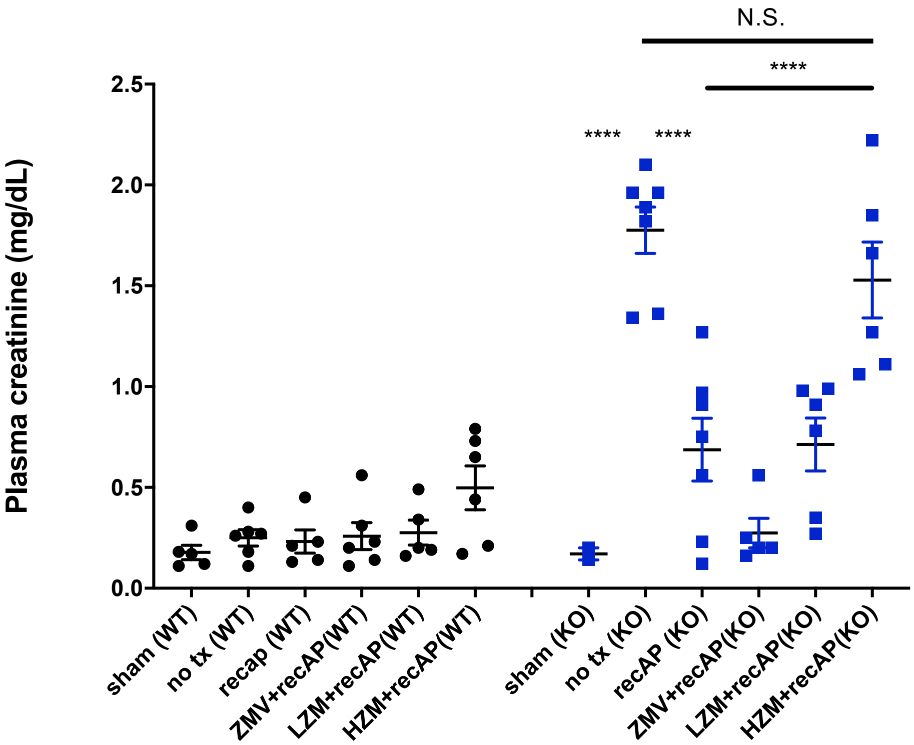

Individual data points for results shown in main Figure 4. WT, C57Bl/6 or $CD73^{+/+}$ littermates (no difference was observed between these 2 groups in response to treatments). KO, $CD73^{-/-}$. Sham, IRI surgery but without clamp. All remaining groups were subjected to 22 min ischemia, and all animals were euthanized 24 hr after IRI surgery/clamp removal. Mice received recAP (2000 U/kg, i.v.) 1 hour before surgery; vehicle for ZM (ZMV; see Methods for vehicle composition) or ZM241,385 (lower dose [LZM; 20 mg/kg] or higher dose [HZM, 100 mg/kg]) was administered s.c. immediately before recAP. ***, p<0.001. ****, p<0.0001.

Supplemental Figure 5. Plasma creatinine in AKI on CKD model in rats prior to randomizing for recAP treatment

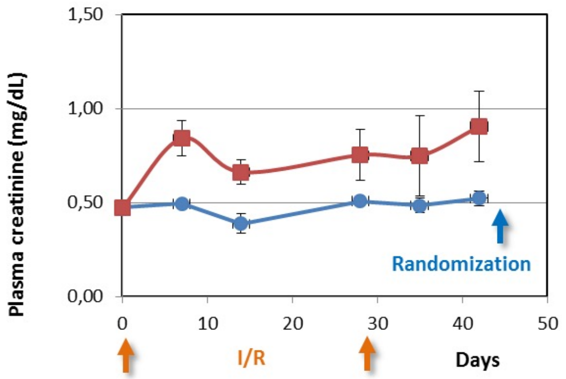

Removal of a single kidney does not affect plasma creatinine, however plasma creatinine increased after successive exposure to periods of ischemia (I/R, red arrows). Sham rats (n=6) underwent a right nephrectomy on day 0 and subsequent sham IRI surgeries (no clamp) on day 0 and 28 (blue circles). CKD rats (n=14) underwent right nephrectomy and 45-minute clamp on left kidney on day 0 then 40-minute clamp on day 28 (red squares). With the exception of day 0, sham and IRI groups at each time point were significantly different from each other ($p < 0.01$). Rats in the IRI group were subsequently randomized for treatment with vehicle or recAP after a third episode of ischemia (see main Figure 6).
